# Genome and transcriptome of the natural isopropanol producer *Clostridium beijerinckii* DSM6423

**DOI:** 10.1186/s12864-018-4636-7

**Published:** 2018-04-10

**Authors:** Hadrien Máté de Gérando, François Wasels, Angélique Bisson, Benjamin Clement, Frédérique Bidard, Etienne Jourdier, Ana María López-Contreras, Nicolas Lopes Ferreira

**Affiliations:** 1 Wageningen Food and Biobased Research, Bornse Weilanden 9, 6709WG, Wageningen, The Netherlands; 2 IFP Energies Nouvelles, 1 et 4 avenue de Bois-Préau, 92852 Rueil-Malmaison, France

**Keywords:** *Clostridium beijerinckii*, DSM6423, Genome, RNA-seq transcriptome, Clustering, IBE fermentation, Isopropanol

## Abstract

**Background:**

There is a worldwide interest for sustainable and environmentally-friendly ways to produce fuels and chemicals from renewable resources. Among them, the production of acetone, butanol and ethanol (ABE) or Isopropanol, Butanol and Ethanol (IBE) by anaerobic fermentation has already a long industrial history. Isopropanol has recently received a specific interest and the best studied natural isopropanol producer is *C. beijerinckii* DSM 6423 (NRRL B-593). This strain metabolizes sugars into a mix of IBE with only low concentrations of ethanol produced (< 1 g/L). However, despite its relative ancient discovery, few genomic details have been described for this strain. Research efforts including omics and genetic engineering approaches are therefore needed to enable the use of *C. beijerinckii* as a microbial cell factory for production of isopropanol.

**Results:**

The complete genome sequence and a first transcriptome analysis of *C. beijerinckii* DSM 6423 are described in this manuscript. The combination of MiSeq and de novo PacBio sequencing revealed a 6.38 Mbp chromosome containing 6254 genomic objects. Three Mobile Genetic Elements (MGE) were also detected: a linear double stranded DNA bacteriophage (ϕ6423) and two plasmids (pNF1 and pNF2) highlighting the genomic complexity of this strain. A first RNA-seq transcriptomic study was then performed on 3 independent glucose fermentations. Clustering analysis allowed us to detect some key gene clusters involved in the main life cycle steps (acidogenesis, solvantogenesis and sporulation) and differentially regulated among the fermentation. These putative clusters included some putative metabolic operons comparable to those found in other reference strains such as *C. beijerinckii* NCIMB 8052 or *C. acetobutylicum* ATCC 824. Interestingly, only one gene was encoding for an alcohol dehydrogenase converting acetone into isopropanol, suggesting a single genomic event occurred on this strain to produce isopropanol.

**Conclusions:**

We present the full genome sequence of *Clostridium beijerinckii* DSM 6423, providing a complete genetic background of this strain. This offer a great opportunity for the development of dedicated genetic tools currently lacking for this strain. Moreover, a first RNA-seq analysis allow us to better understand the global metabolism of this natural isopropanol producer, opening the door to future targeted engineering approaches.

**Electronic supplementary material:**

The online version of this article (10.1186/s12864-018-4636-7) contains supplementary material, which is available to authorized users.

## Background

The use of petrochemical derived fuels and chemicals such as olefins cause severe damages to the environment and is not sustainable in the long term, due to the limited nature of fossil oil. Approaches based on the conversion of renewable biomass into chemicals of interest may represent a sustainable alternative to replace, at least partially, these petroleum-based chemicals. As an example, isopropanol can be added into fuel blends [1] or converted into olefins using chemical dehydration steps [2]. Isopropanol is a commercial C3 alcohol used as solvent or antifreeze, which can also be used as a precursor of propylene, one of the main platform molecules used in the chemical industry. Very few microorganisms have been described as natural isopropanol producers and almost none of them has been thoroughly studied. Solventogenic *Clostridia* have been mainly studied for their ability to produce a mixture of acetone, butanol and ethanol (ABE) from a variety of substrates (including C6 and C5 sugars), and a few strains are also able to further reduce acetone into Isopropanol, yielding an Isopropanol, Butanol and Ethanol (IBE) mixture. *C. acetobutylicum* ATCC 824 and *C. beijerinckii* NCIMB 8052 are recognized as model organisms for ABE production, while *C. beijerinckii* DSM6423 (formerly NRRL B-593) is the best known IBE producer. The genome of *C. beijerinckii* NCIMB 8052 was sequenced a few years ago and consists in a 6.0 Mb chromosome [3], which is 50% larger than that of *C. acetobutylicum* ATCC 824 [4]. Unlike these strains, the assembled genome of *C. beijerinckii* DSM 6423 (NRRL B-593) is not available, even if it has been sequenced through a Hiseq approach recently, together with 29 other Clostridial strains, in order to perform a comparative genomic analysis of saccharolytic strains belonging to the genus of *Clostridium* [5].

Transcriptional analysis is mandatory to gain knowledge about gene regulation and thus determine strategies for strain improvement. To date, transcriptomic analyses of solventogenic *Clostridia* have focused on ABE strains [6]. Most of these transcriptomic analyses were performed using DNA microarray methods and other hybridization techniques exhibiting a relatively low dynamic range for the detection of transcriptional levels due to background, saturation and poor sensitivity for gene expression. RNA-seq has now supplemented these approaches because of its capacity to provide a more accurate quantification and a larger dynamic range of expression levels. In addition, it has very low background noise because DNA sequence reads can be unambiguously mapped to unique regions along the genome [7]. The genome-wide transcriptional dynamics of *C. beijerinckii* NCIMB 8052 over a batch fermentation process was investigated using these technique [8].

This study presents the complete genome sequencing of the currently most studied natural isopropanol producing strain, together with RNA-seq analyses covering its whole fermentation metabolism for glucose conversion into IBE. Comparative analysis with the model organisms highlights some transcriptomic regulations.

## Methods

### Bacterial culture and bioreactor scale fermentations

Laboratory stocks of *C. beijerinckii* DSM 6423 spores were stored in sterile H_2_O at − 20 °C. Spores were heat shocked in boiling water for 1 min, then inoculated at a 2% inoculum level into 50 mL medium containing per liter: yeast extract, 5.0 g; KH_2_PO_4_, 0.75 g; K_2_HPO_4_, 0.75 g; MgSO_4_·7H_2_O, 0.4 g; MnSO_4_·H_2_O, 0.01 g; FeSO_4_·7H_2_O, 0.01 g; NaCl, 1.0 g; asparagine, 2.0 g; (NH_4_)_2_SO_4_, 2.0 g; cysteine, 0.125 g; and glucose 12.5 g. The medium was incubated at 37 °C ± 1 °C for 24 h in an anaerobic environment under N_2_:CO_2_:H_2_ (volume ratio of 85:10:5) atmosphere. Subsequently, the actively growing culture was inoculated at 5% into a fermentation medium (GAPES [9]) containing per liter: yeast extract, 5.0 g; KH_2_PO_4_, 1.0 g; K_2_HPO_4_, 0.76 g; NH_4_Ac, 3.0 g; MgSO_4_·7H_2_O, 1.0 g; FeSO_4_·7H_2_O, 0.1 g; *p*-aminobenzoic acid, 0.1 g; and glucose, 60 g; in a 500 mL bioreactor system as described previously [10]. Temperature was controlled at 37 °C ± 1 °C. A stirring at 350 rpm was employed for mixing. Cell density and product concentration were monitored through the course of fermentation.

### Experimental design

In order to get 3 biological replicates, the same procedure was repeated 3 times on 3 different weeks. Each week, a fresh preculture was used to inoculate 2 identical bioreactors. Samples were taken over the early exponential, late exponential and stationary phases (samples at 3, 6, 8, 11, and 24 h). Following centrifugation of the samples, cell pellets were immediately frozen in liquid nitrogen and supernatants were used for HPLC analysis. For each timepoint, the RNA samples of the 2 bioreactors were pooled, leading to 1 RNA sample per bioreactor and timepoint.

### Culture growth and fermentation products analysis

Culture growth was measured by following optical density at 600 nm (OD600) in the fermentation broth using an Ultrospec 2000 (Pharmacia Biotech) spectrophotometer. Metabolites were determined in clear supernatants of samples taken from the fermentation. Sugars, solvents, and organic acids were determined by HPLC using a gel permeation/size exclusion column (Shodex Ionpack KC-811) coupled to a refractometer and UV detector as described earlier [10, 11].

### Genomic DNA extraction and sequencing

Genomic DNA of *C. beijerinckii* DSM6423 was purified using the GenElute bacterial genomic DNA kit (Sigma-Aldrich, Saint-Louis, USA). Concentration of gDNA was determined using Qbit spectrophotometer (Thermofisher) and quality checked on 0.5% agar gel. Mi-seq analysis was performed by the I2BC plat-form (Saclay, France) using the following protocol. Genomic DNA samples was fragmented using a “‘COVARIS S2” sonicator, to a mean size of 700 bp. DNA Libraries were constructed from 1 μg of this fragmented DNA, according to the “Illumina DNA sample prep protocol” (End-repair, A-tailing, ligation, PCR enrichment), on a Beckman SPRI-TE automate and using reagents from the “SPRIworks Fragment library system I” kit (Beckman Coulter). The final product were gel purified to achieve a library mean size of 700 bp. Library quality was assessed on an agilent Bioanalyzer instrument (Agilent) and sequenced on a Illumina MiSeq instrument, using a Paired-end 2 × 250 bp protocol, with a MiSeq Reagent Kit v2 500 cycle (MS-102-2003, Illumina), according to the constructor recommendations. The run resulted in a 80× coverage of the genome of *C. beijerinckii* and the different reads, spanning 250 nucleotides each, were assembled into DNA fragments called contigs. De novo sequencing was performed using the PacBio RSII platform (GATC, Germany).

Contigs were uploaded to the MicroScope bioinformatics platform where the putative genes were automatically annotated through a specific computer workflow [12]. MicroScope is a Microbial Genome Annotation & Analysis Platform developed by the Genoscope institute (Evry, France) [13]. Sequenced strains and RNAseq results can be uploaded and automatically analyzed on this platform.

### RNA extractions and RNA-seq protocols

The extraction was performed as previously described [8]. Briefly, cell lysis and RNA isolation was done with the TRIzol Plus RNA purification kit (AMbion). 5 mL TRIzol reagent was added to the frozen pellet followed by 1 mL chloroform after cell lysis. After shacking and centrifugation, the upper phase was mixed with an equal volume of 70% ethanol before transfer to PureLink RNA Spin Cartridges furnished with the kit. Kit instructions allowed to obtain purified total RNA which was further processed by cleanup with the RNeasy mini kit (Qiagen). DNA was eliminated with DNAse I treatment (AM1906, Invitrogen). 20 μL RNA were treated with 2 μL DNAse in 50 μL nuclease free water for 30 min at 37 °C before addition of another 1.5 μL DNAse and 30 min extra incubation. Treatment was done twice and was followed by RNA cleanup with the RNeasy kit. A full quality assessment of the RNA, quantification using Nanodrop, PCR to check for residual DNA and migration on 1% agarose gel were done before depletion of ribosomal RNA with MicrobExpress kit (ThermoFischer). Depleted RNA samples were quantified and stored at − 80 °C before being sent for analysis. DNA libraries from RNA samples and Hi-seq single read sequencing were performed by the Imagif platform (I2BC, Orsay, France).

### RNA-seq sequencing data analysis

Clean reads of each sample were mapped to the reference genome of the previously sequenced *C. beijerinckii* DSM 6423, with predicted protein encoding genes and rRNA using the TAMARA (Transcriptome Analysis based on MAssive sequencing of RnAs) tool provided on the MicroScope platform [12]. Table 1 shows statistics about the number of total and mapped reads.

**Table 1:** Summary of RNA-Seq sequencing and data analysis results

Replicate	Time point (h)	Total read number	Reliable reads	Reads mapped on genomic objects (except rRNA)	Number of genes with detectable expression
A	3	19,158,892	17,799,816	7,024,274	5944
B	3	11,574,716	10,099,244	3,266,941	5829
C	3	15,203,792	12,936,903	4,403,183	5851
A	6	16,007,459	14,775,252	5,341,913	5876
B	6	13,731,477	11,723,511	4,255,219	5884
C	6	13,472,975	11,754,891	3,741,547	5861
A	9	11,050,565	9,196,745	2,854,832	5895
B	9	14,381,148	12,261,616	3,777,589	5906
C	9	11,873,306	10,118,493	3,235,171	5880
A	11	13,663,240	11,531,714	3,327,568	5887
B	11	10,195,910	8,792,217	2,208,510	5789
C	11	19,046,554	16,439,661	4,596,207	5917
A	24	13,578,705	11,371,420	1,153,741	5203
B	24	21,453,820	17,646,890	1,871,469	5606
C	24	9,805,052	8,262,117	1,046,981	5121

Transcriptomic high throughput sequencing data were analyzed using a bioinformatic pipeline implemented in the Microscope platform [12]. In a first step, the RNA-Seq data quality was assessed by including reads trimming. In a second step, reads were mapped onto the *C. beijerinckii* DSM6423 genome sequence (previously submitted for sequencing) using the SSAHA2 package [14] that combines the SSAHA searching algorithm (sequence information is encoded in a perfect hash function) aiming at identifying regions of high similarity, and the cross-match sequence alignment program [15], which aligns these regions using a banded Smith-Waterman-Gotoh algorithm [16]. An alignment score equal to at least half of the read is required for a hit to be retained. To lower false positives discovery rate, the SAMtools (v.0.1.8, [17]) are then used to extract reliable alignments from SAM formatted files. The number of reads matching each genomic object harbored by the reference genome is subsequently computed with the Bioconductor GenomicFeatures package [18]. If reads matching several genomic objects, the count number is weighted in order to keep the same total number of reads. Finally, the Bioconductor-DESeq package [19] with default parameters is used to analyze raw counts data and test for differential expression between conditions. Moreover, the quality of the RNA-seq data was strengthened by checking the relative stability of housekeeping genes such as *gyr*A and *rpo*B across time points.

## Results

### Genome assembly

**Fig. 1:**
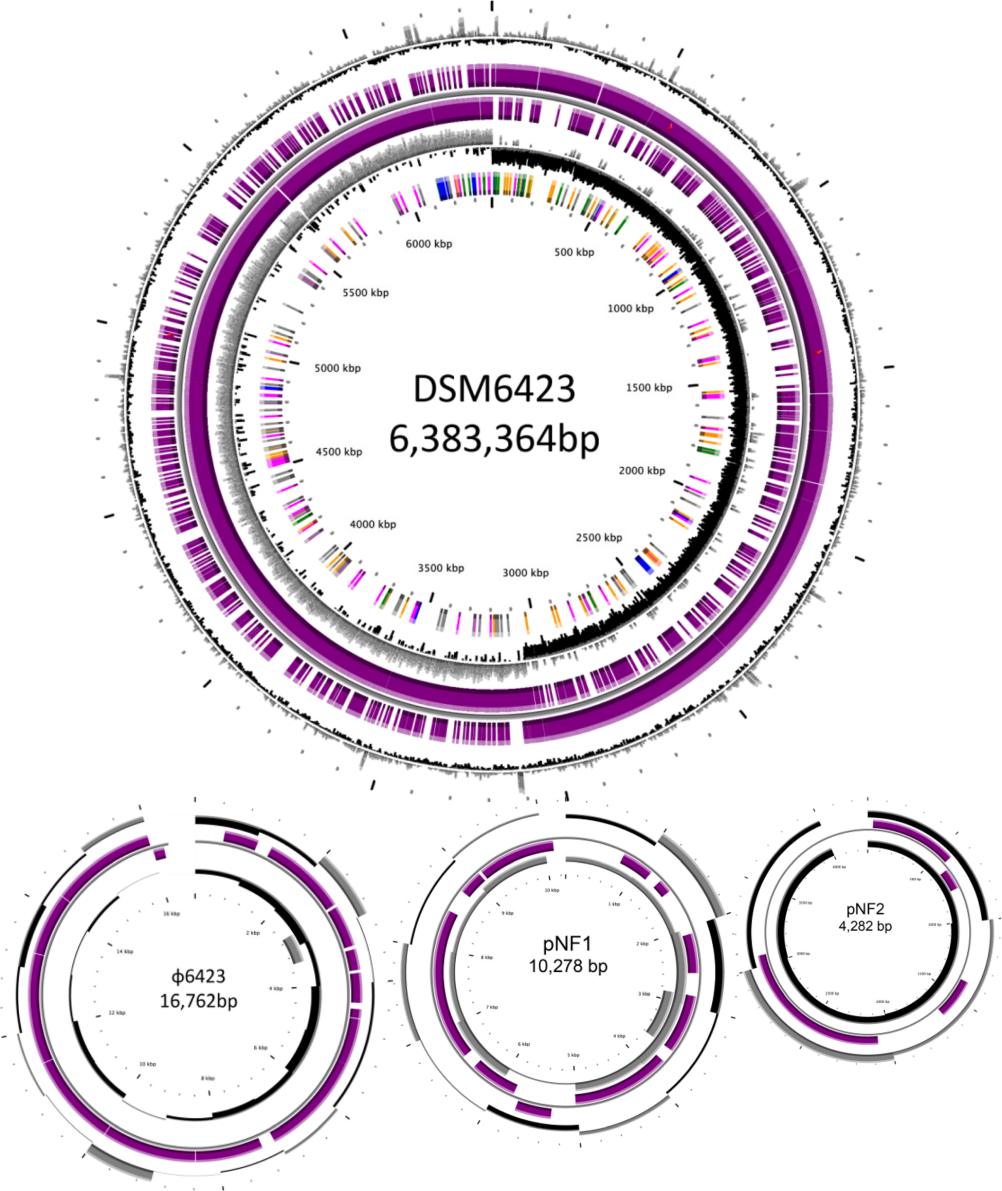
Circular representation of *Clostridium beijerinckii* DSM6423 genome. CGView representation [51] of the circular genome of the DSM6423 strain and its extrachromosomic elements (plasmids: pNF1, pNF2 and double stranded linear phage ɸ6423). Circles display (from the outside): (1) GC percent deviation (GC window - mean GC) in a 1000-bp window. (2) Predicted CDSs transcribed in the clockwise direction. (3) Predicted CDSs transcribed in the counterclockwise direction. Genes displayed in (2) and (3) are color-coded according different categories: red and blue: MaGe validated annotations orange: MicroScope automatic annotation with a reference genome purple: Primary/Automatic annotations. (4) GC skew (G + C/G-C) in a 1000-bp window. (5) rRNA (blue), tRNA (green), misc_RNA (orange), Transposable elements (pink) and pseudogenes (grey)

Genome sequence of *C. beijerinckii* DSM6423 was obtained through PacBio and MiSeq sequencing. The PacBio de novo sequencing yielded 17 unitigs. Unitigs were further combined in four scaffolds when assembled with the 412 contigs obtained through MiSeq sequencing. Junctions between unitigs were confirmed by PCR, and the 4 scaffolds were PCR gap-closed, yielding a curated 6,383,364-bp long circular chromosome, with an average GC content of 29.81% (Fig. 1). Automatic functional annotation resulted in the prediction of 6052 protein genes and 202 RNA genes on this chromosome.

Three Mobile Genetic Elements (MGE) were also identified: two natural plasmids named pNF1 and pNF2, and one bacteriophage, named ɸ6423 (Fig. 1). Plasmid pNF1 is 10,278-bp long and contains a gene sharing homology with both the bacteriocin gene from *Clostridium butyricum* MIYAIRI 588 [20] and the closticin 574 gene from *Clostridium tyrobutyricum* ADRIAT 932 [21]. The last 83 aminoacids of the gene product shared 81.9% and 64.6% pairwise identity with the corresponding peptides possessing bacteriocin activity from *C. butyricum* MIYAIRI 588 and *C. tyrobutyricum* ADRIAT 932, respectively. Plasmid pNF2 is 4282-bp long and carries four predicted ORFs, one of them being potentially involved in plasmid replication. Phage ɸ6423 have a linear double-stranded DNA genome of 16,762 bp, with 143 bp inverted terminal repeats. To our knowledge, this is the first time such a bacteriophage is described in the *Clostridium* genus.

Identification of Regions of Genomic Plasticity (RGPs) was performed on the DSM6423 chromosome in order to identify potentially horizontally transferred genes (HGT) which are gathered in genomic regions. In total, 47 RGPs were found in the genome of DSM 6423 when compared with that of *C. beijerinckii* NCIMB 8052. One of these RGPs contains the specific secondary alcohol dehydrogenase *adh* gene whose product allows the conversion of acetone into isopropanol. This gene is located in a 23-kb genomic island having no equivalent in the NCIMB 8052 strain and harboring 28 coding sequences in which 6 metabolic genes and one transposase were predicted (Additional file 1). There is a high probability that this part of the genome was acquired through horizontal transfer. This NADP(H)-linked, zinc-containing secondary alcohol dehydrogenase (sADH) is a tetrameric protein having the highest catalytic efficiency on acetone [22, 23]. Interestingly, This sADH has a significant sequence identity with other class I alcohol dehydrogenases such as the tetrameric enzymes from *Entamoeba histolytica* and bacterial secondary-alcohol dehydrogenase from *Thermoanaerobacter brockii* which is an exceptionally high degree of identity between enzymes from such distantly related organisms.

### *C. beijerinckii* cultivation in bioreactor

**Fig. 2:**
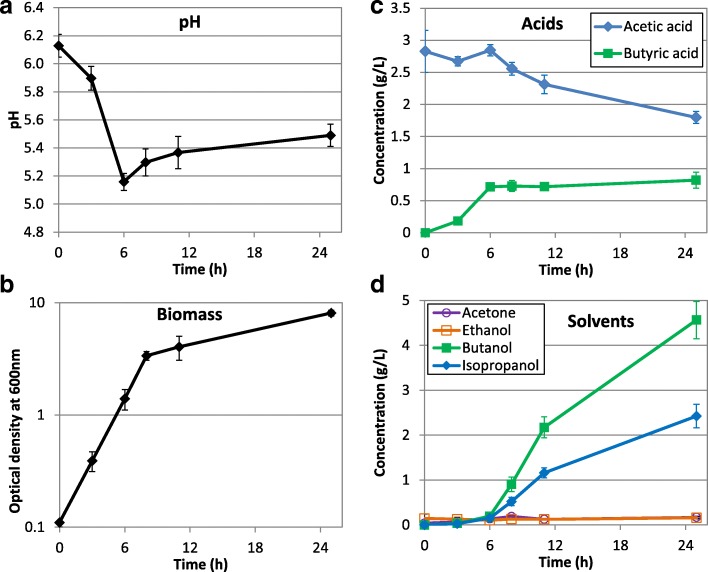
Fermentation profile of *Clostridium beijerinckii* DSM6423 on glucose. *C. beijerinckii* DSM 6423 was cultivated in bioreactors in GAPES medium. **a** pH, **b** biomass followed by OD_600_, **c** acids and **d** solvents. Values are the mean and standard deviation of the 6 biological replicates. See Additional file 3 for details on the biological replicates

To further improve genetic knowledge on *C. beijerinckii* DSM6423, a transcriptomic study was performed. A RNA-Seq approach was chosen in order to have a timelapse study of the metabolism of the strain throughout the fermentation process. Three independent fermentations were carried out in bioreactors on three different weeks, showing good reproducibility (Fig. 2).

Acid re-assimilation and solvent production appeared to start around 6-8 h. This was concomitant with the characteristic loss of mobility of the cells and the appearance of cigar–shape cells as observed under the microscope (Additional file 2).

The final solvent profile obtained corresponded well to those described earlier for this strain [24, 25]. Moreover, the biphasic pH is characteristic of the solvantogenic strains such as the well-known *C. acetobutylicum* ATCC824 [26], even if the latter is able tolerate more acidic pH conditions. In the presence of sodium acetate in the medium, *C. beijerinckii* DSM6423 starting its switch to solvantogenesis at a higher pH level (5,2) than *C. acetobutilicum* ATCC824 (pH 4,2 [27]). Survase et al. [24] studied the time course of a batch fermentation on glucose and showed that solvent production started at the end of the exponential phase, at approximately 6–9 h, which is consistent with our observations. Their batch culture showed a maximum concentration of 2.16 g/L (36.8% of total solvents) of isopropanol and 3.71 g/L (63.2% of total solvents) of butanol after 24 h fermentation with no further significant increase and the maximum solvent yield was 0.30 g/g glucose consumed with 33.8% of substrate conversion. Interestingly, the Isopropanol/Butanol ratio was progressively growing along the solvantogenic phase (Fig. 2a). This may be due to the presence of sodium acetate in the fermentation medium to prevent a severe pH drop linked to acids transport and assimilation [28]. This behavior is in contradiction with those observed for a batch culture of *C. beijerinckii* DSM6423 with a control of the pH (close to 4.8) using NaOH [24]. In that case, a constant I/B ratio was maintained during the ten first hours of solvantogenesis. Interestingly, in the case of *C. acetobutylicum*, [29] a significant increase of ABE production but no modification of the A/B ratio was observed when ammonium acetate is used to control the pH. This tend to confirm a specific behavior of *C. beijerinckii* DSM6423.

During our batch fermentation we obtained 3 g/L isopropanol and 5.3 g/L butanol after 30 h fermentation time with 28.8% substrate conversion (Additional file 3). De Vrije et al. reported end concentrations of 10.7 g/L total IBE, with 3.2 g/L isopropanol and 6.9 g/L of butanol after 48 h of fermentation when the strain was grown on a mix of glucose and xylose [30].

For each triplicate, samples were collected for RNA-Seq analyses at 3 h (acidogenesis phase, exponential growth), 6 h (acidogenesis to solventogenesis switch), 8 h and 11 h (solventogenesis) and 24 h (late solventogenesis) after inoculation. The 15 resulting RNA samples were sequenced and analyzed using the reconstructed genome of DSM6423.

### RNA seq analysis

#### Highly expressed genes

Determination of the most highly expressed genes was performed using RPKM values during the whole fermentation experiment (Additional file 4). About 15% of these genes were coding for proteins of unknown functions, which represent 34% of the coding sequenced predicted in the genome. As expected, the most expressed genes after 3 h of fermentation are coding for ribosomal proteins, consistently with the protein synthesis activity during exponential growth phase and cell division. A set of genes involved in cell motility, such as the flagellin coding protein, was also found to be highly expressed, consistently with the high mobility of the *Clostridium* cells at this time (Additional file 2). Other highly expressed genes at 3 h were involved in transcription, translation, protein folding and of course Isopropanol/Butanol/Ethanol production. Concerning the mobile genetic elements discovered in *C. beijerinckii* DSM6423, 3 highly expressed genes were located on the bacteriophage Φ6423 and only one, corresponding to a cell wall binding protein, on the natural plasmid pNF1.

#### Differentially expressed genes

**Fig. 3:**
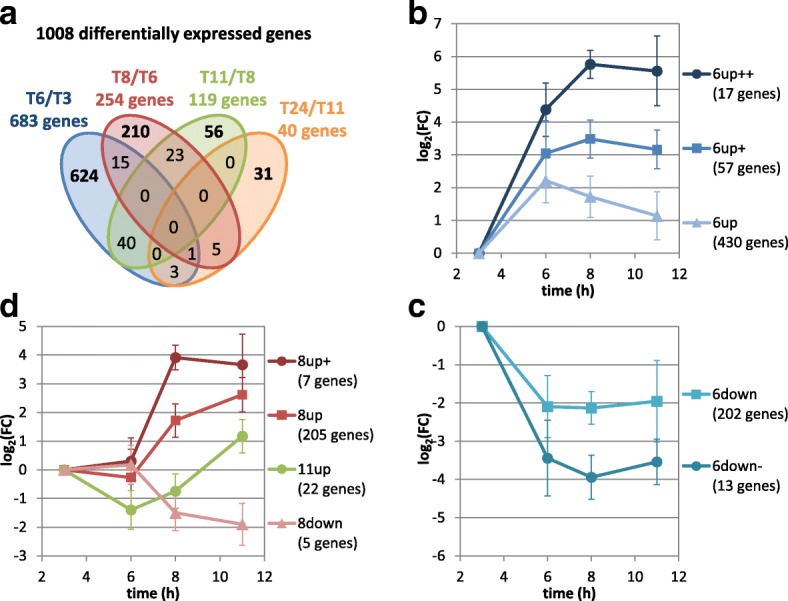
Global transcriptomic analysis of *C. beijerinckii* DSM6423 fermentation on glucose. **a** Venn Diagram showing the number of genes regulated in various physiological time points. **b** to **d** kinetic expression profiles of various clusters of genes: genes up-regulated at 6 h (B), genes down-regulated at 6 h (**c**), and genes regulated at 8 h or 11 h (**d**)

In order to investigate the transcriptional regulations during the fermentation, the most differentially expressed genes were selected by comparing their expression at a given time point (6 h, 8 h, 11 h and 24 h) to their expression at the previous time point (3 h, 6 h, 8 h and 11 h respectively) (Fig. 3, a). Thresholds were selected at |log_2_(fold change)| > 1.5 and adjusted *p*-value< 0.001, which resulted in a list of 1008 significantly differentially expressed genes (16% of the genome). Interestingly, the majority of these genes (683 genes) showed a different level of gene expression between 3 h and 6 h, which corresponds to the switch from acidogenesis to solventogenesis. Most of the genes (938 genes, 93%) were regulated once during the fermentation, 69 genes were regulated 2 times, and only 1 gene appeared to be regulated 3 times during the fermentation. This tends to demonstrate that each time point corresponded to a specific physiological state during the cultivation As suggested by the number of regulated genes, the main transcriptomic regulations occur between 3 and 11 h, with only 31 genes being specifically regulated between 11 and 24 h. Therefore, further clustering analysis focused on the 977 genes regulated between 3 and 11 h.

### Kinetic analysis of transcription regulations

In order to highlight the transcriptional regulation along the fermentation, we calculated the transcription profiles of each gene, using the 3 h sample as reference. Clustering was performed using CAST algorithm [31] with a threshold of 0.88 and revealed 8 clusters containing 953 genes. Three clusters (504 genes) correspond to genes up-regulated at 6 h (Fig. 3b), 2 clusters (215 genes) to genes down regulated at 6 h (Fig. 3c) and 3 clusters (234 genes) to genes up-regulated at 8 h or 11 h (Fig. 3d).

Up-regulated genes at 6 h are divided in 3 different clusters (Additional file 5). A first cluster (6up++) contains 17 genes up-regulated at 6 h and 8 h and involved in the Panthothenate and CoA biosynthetic pathway, oxidative stress or solvantogenesis (butanol production). A second cluster of 57 genes (6up+), strictly up-regulated at 6 h, comprises a set of genes related to sporulation, including the predicted anti-sigma F factor antagonist (SpoIIAA and SpoIIAB), and a putative operonic structure linked to acetone production (CIBE_4606 to 4609).

The third cluster (6up) comprises 430 genes that are up-regulated at 6 h and then down-regulated. This cluster include genes that are not regulated after 6 h, notably the anti-sigma factors regulating sporulation, an acetolactacte synthase and several genes encoding for enzymes involved in ferrous storage (ferritin). Some genes of this cluster are directly down-regulated at 8 h, including genes involved in solvantogenesis (isopropanol production), responses to the heat shock effects and suffer assimilation. Finally, several genes of this cluster are down-regulated at 11 h including sigma factors related to the initiation of sporulation and a RNA polymerase sigma factor 28. The σ28 is a minor sigma factor responsible for the initiation of transcription of a number of genes involved in motility and flagellar synthesis [32].

The 235 genes that were down-regulated at 6 h were divided in two different clusters. The main one (6down) contained a gene cluster involved in the mobility of the bacterial cells, in particular *motA* and *motB*. This is in agreement with the loss of mobility and the change in cell shape characterizing the onstart of the solventogenic phase (Additional file 2). The second cluster of genes, down-regulated at 6 h then 8 h (6down-) corresponded to genes coding for ribosomal proteins, oxidative stress response proteins and a high number of putative membrane transporters. This might suggest some major changes in the membrane transport system for the re-assimiliation of acids linked to solventogenesis.

The set of genes up-regulated after 8 h was divided in 3 different clusters including a first one (8up, 205 genes) mainly comprising genes involved in solvantogenesis. The second cluster (8up+) of genes was strictly up-regulated at 8 h and included genes linked to oxidative stress and the latter phases of sporulation. The last cluster (11up) corresponded to a set of 22 genes up-regulated at 11 h and mainly linked to sporulation.

A really small cluster of genes were down regulated at 8H (8down). Among them, one gene is coding for a member of the 2-oxoacid oxidoreductases, a family of enzymes that oxidatively decarboxylate different 2-oxoacids to form their CoA derivatives. Moreover, a putative operonic structure classified in this cluster is potentially involved in ferrous iron transport (CIBE_5053 to 55).

### Main transcriptomic regulations

#### Central metabolism

In order to better understand the transcriptional regulation occurring in *C. beijerinckii* DSM6423 cells during fermentation, the transcriptomic data allowed us to confirm the role of some predicted metabolic genes coming from the automatic annotation. Analysis revealed 22 genes potentially involved in glycolysis, with multiples candidates (“isozymes”) for some key reactions. A majority of these genes was highly transcribed throughout the fermentation tending to demonstrate their implication in this central pathway.

**Fig. 4:**
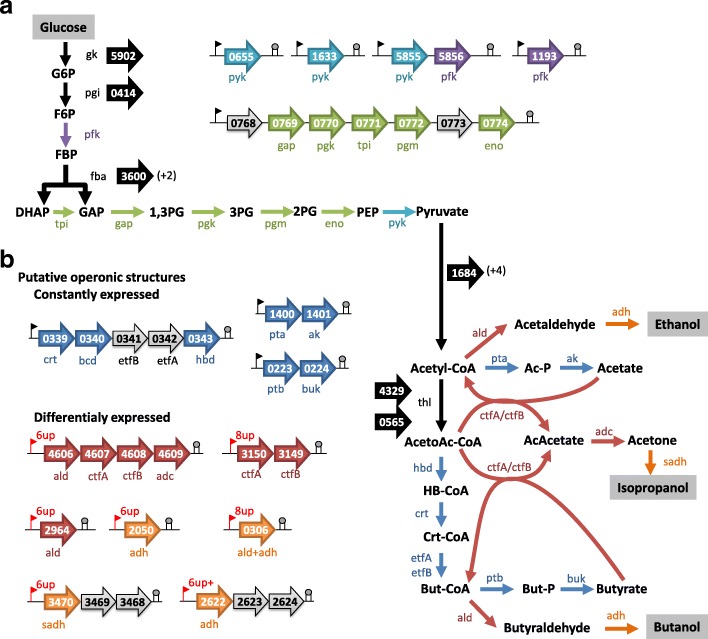
Main genes and predicted operonic structures involved in the central metabolism of in *C. beijerinckii* DSM6423. **a** glycolysis; **b** acids and solvents production). Number of isozymes, predicted by Microscope tool (Genoscope, Evry, France) are indicated in brackets

Transcriptomics analysis of the whole metabolic pathways also revealed a significant expression level of predicted operons encoding enzymes involved in the production of acids and the conversion of acetyl-CoA to butyryl-CoA (Fig. 4). No regulation was observed for operonic structures predicted to be involved in acetate (CIBE_1400 and 1401) or butyrate (CIBE_0223 and 0224) production. This might be explained by the specific medium used in these experiment, containing 3 g/L of sodium acetate. Unlike what has been observed in *C. acetobutylicum* (Schreiber & Dürre, 2015), the gene coding for the phosphoglycerate mutase (*pgm*, CIBE_0772) is predicted to be a part of the operon comprising genes coding for the glyceraldehyde-3-phosphate dehydrogenase (*gap*), the phospho-glycerate kinase (*pgk*) and the triosephosphate isomerase (respectively *gap*, *pgk* and *tpi,* CIBE_0769 to 0771; Fig. 4).

As observed in other *Clostridium* strains, genes dedicated to the production of butyryl-CoA from acetoacetyl-CoA seems to be organized in an operon (CIBE_0339 to 0343, Fig. 4) and are not regulated in our fermentation conditions. More interestingly, the 3 genes (CIBE_5186, CIBE _2196 and CIBE _1684) apparently involved in the pyruvate decarboxylation into acetyl-CoA do not appear to be regulated in the same way. The first one was highly and constantly transcribed throughout the fermentation whereas the two others were slightly transcribed (30× less) and further induced at 8 h and 11 h. This might suggest a fine-tuned regulation of the acetyl CoA production along the fermentation.

Another operon containing CIBE_4606 to CIBE_4609 gene, coding for an alcohol dehydrogenase, two CoA transferases and an acetoacetate decarboxylase, are equivalent to the *ald-ctfA-ctfB-adc* operon of *C. beijerinckii* NCIMB 8052 [3]. Expression of this operon appeared stable throughout the fermentation. Two genes encoding *ctfA* and *ctfB* analogs (CIBE_3149 and 3150) were then identified and appeared to be overexpressed at 8 and 11 h.

More surprisingly, a high level of transcription was already observed at 3 h for the predicted operons involved in butanol (CIBE_2622 to CIBE_2624), acetone (CIBE_4606 to CIBE_4609) or isopropanol (CIBE_3468 to CIBE_3470; Fig. 4) production. Expression of this latter operon was even up-regulated at 6 h. This specificity confirm the distinct transcriptomic regulation of the solvantogenesis between *C. beijerinckii* and *C. acetobutylicum* strains. In *C. acetobutylicum* ATCC824, the corresponding genes adhE (CA_P0162), ctfA (CA_P0163) and ctfB (CA_P0164) are located on the mega-plasmid pSOL1 and only induced during solvantogenesis, while adc (CA_P0165) is organized in a monocistronic operon in the opposite direction [33, 34]. The switch between acidogenic and solventogenic phases appears to be less pronounced in the case of *C. beijerinckii* DSM6423, compared to other solventogenic clostridia, such as *C. acetobutylicum* ATCC824. In total, more than 20 genes predicted as encoding alcohol dehydrogenases were detected in the genome of the DSM 6423 strain and most of them were expressed from the beginning of the fermentation.

#### Sporulation

It is well established that *spo0A* is the master regulator for sporulation events in *Bacillus* and in *Clostridia* [35, 36]. Phosphorylated Spo0A has been reported to induce the expression of several targets, including the sol operon and multiple sporulation sigma factor genes in *C. acetobutylicum* ATCC 824 [37]. As described previously, the solventogenic phase started at 6 h and may be linked to the initiation of the sporulation phase. This was confirmed by the overexpression at 6 h of a set of 12 genes including the predicted genes coding for Spo0A (CIBE_2041; Additional file 5), the sigma factors σF (CIBE_0994) and σG (CIBE_1357) but also the anti-anti-sigma factor SpoIIAA and the anti-sigma factor SpoIIAB (CIBE_0992 and 0993, respectively). In *C. acetobutylicum* ATCC824, a multicomponent system involving SpoIIAA and SpoIIAB activates Spo0A through phosphorylation, which further stimulates the expression of sigF [38]. The overexpression at 6 h of the predicted anti-sigma factor SpoIIAB and the specific regulation of the SpoIVB predicted gene (repressed at 6 h then up-regulated at 8 h; Additional file 5) suggest that the initiation of the sporulation and solventogenesis are simultaneous.

An increase in the expression of some other sporulation-related genes, such as spoIVA and spoIVB was also detected at 8 h. In *Bacillus subtilis*, SpoIVB protein is the key determinant for inter-compartmental signaling of pro-σK processing [39, 40]. Pro-σK is the inactive form of the final transcription factor σK acting in the mother cell compartment of the sporulating cell [41]. The σK regulon is then involved in the final stages of spore maturation including spore coat biosynthesis and release of the spore from the sporangial cell. This is in accordance with the observation that these genes are up-regulated at 8 h.

#### The specific regulation of the motility locus

Loss of motility usually comes with the transition from exponential to stationary growth phases and therefore when switching from acidogenesis to solventogenesis. *Clostridia* cells stop moving and lose their rod shape to gain the characteristic cigar shape instead. Expression of motility genes, such as *motA* and *motB*, and of a high number of genes coding flagellar proteins was repressed at 6 h, corresponding to the start of the solventogenic phase. This is in accordance with previous transcriptomics results observed in *Clostridium beijerinckii* NCIMB8052 [8], in *Clostridium acetobutylicum* [42] and more generally in Clostridia and Bacilli [43]. Most genes in the flagellar/chemotaxis cluster were down-regulated at the beginning of the solvantogenesis, which is related in our strain with the onset of sporulation. Similarly, the flagellar motility coding gene *flgC* is strongly expressed during the early exponential phase and further down regulated at 6 h, at the onset of the stationary phase.

## Discussion

*C. beijerinckii* is a prominent solvent-producing bacterium with great potential as a microbial cell factory for the biofuel and chemical industries, in particular for isopropanol production. This strain has already been used for advanced bioprocesses, and increased productivities were obtained by cell immobilization, in-situ product removal and use of glucose/xylose mixes as substrate [44]. Recently, mutagenesis and genome shuffling have been applied to this strain to generate mutants with improved tolerance to isopropanol [11]. However, a complete genome characterization of this strain was still lacking.

Although transcriptional analysis is essential to understand gene functions and regulation and thus elucidate proper strategies for further strain improvement, limited information was available for the natural isopropanol producer *C. beijerinckii* DSM 6423. The genome sequencing of DSM 6423 yielded a 6,4 Mbp chromosome, two plasmids and one bacteriophage. These mobile genetic elements could be used as a chassis for the development of genetic tools dedicated to this strain.

The genome-wide transcriptional dynamics of *C. beijerinckii* NCIMB8052 revealed an overexpression of the glycolysis genes throughout the fermentation, especially during the acidogenesis phase. The expression of genes involved in this first part of the metabolism was then down-regulated at the beginning of the well-known metabolic shift from acidogenesis to solventogenesis. According to Wang et al. [8], out of the > 20 genes encoding alcohol dehydrogenase in *C. beijerinckii* NCIMB8052, Cbei_1722 and Cbei_2181 were highly up-regulated at the onset of solventogenesis, corresponding to their key roles in primary alcohol production. In our fermentation experiments, the expression pattern of glycolysis genes was similar than in *C. beijerinckii* NCIMB8052, with high expression levels throughout the whole fermentation [8]. This seems to be specific of *C. beijerinckii* strains as a time course transcriptional analysis of *C. acetobutylicum* ATCC824 showed higher expression during stationary phase for most of the glycolysis genes [35].

Studies concerning *C. acetobutylicum*, with genome-wide gene expression analysis of the switch between acidogenesis and solventogenesis [42] or solvent stress response using butanol [6] led to new insights into the physiological role of several genes or operons involved in solvent formation. Concerning *C. beijerinckii* DSM 6423, using the MicroScope web-platform to identify the genes involved in the main metabolic pathways and their duplications, we were able to detect at least 4 putative operonic structures related to acidogenesis and solvantogenesis. One of them, CIBE_4606 to 4609, corresponds to the sol operon of *C. beijerinckii* NCIMB8052 where *ald*, *ctfAB* and *adc* are organized in an operon and co-regulated [8]. The structure of other important gene operons involved in metabolic pathways for acid and solvent production in *C. beijerinckii* NCIMB 8052, including *pta*-*ack*, *ptb*-*buk*, *hbd*-*etfA*-*etfB*-*crt* operons was also confirmed.

Most sporulation genes in *C. beijerinckii* NCIMB8052 demonstrated similar temporal expression patterns to those observed in *B. subtilis* and *C. acetobutylicum*. Sporulation sigma factor genes *sigE* and *sigG* exhibited accelerated and stronger expression in *C. beijerinckii* NCIMB8052, which is consistent with the more rapid forespore and endspore development in this strain. A previous study involving insertional inactivation of *spo0A* indicated that Spo0A does control the formation of solvents, spores and granulose in *C. beijerinckii* NCIMB8052 [45]. A *spo0A* mutant of *C. beijerinckii* NCIMB8052 also showed an asporogenous and non-septating phenotype [46]. Moreover, in *C. acetobutylicum* ATCC 824*, spo0H* and *spo0A* are both constitutively expressed at constant levels throughout the growth cycle [36]. In our strain, production of solvents was detected already at 6 h after inoculation of the fermenters and initiation of sporulation was concomitant with the mid to late solventogenic phase (8 to 11 h). There is a slight down-regulation of *spo0A* at 8 h, similar to what Wang et al. observed with NCIMB8052 [8], and microscopic studies indicated that sporulation was both enhanced and accelerated due to *spo0A* overexpression compared to parental strains. It is therefore not surprising to see sporulation genes, such as *spoIVA*, highly expressed starting at 11 h.

The expression of motility genes, such as *motA* and *motB*, and flagellar protein coding *flgC* was repressed at 6 h, which corresponds to the onstart of the solventogenic phase in the studied fermentation. In *Salmonella enterica,* electrostatic interactions between the stator protein MotA and the rotor protein FliG are important for bacterial flagellar motor rotation [47]. The Salmonella flagellar motor, which is embedded within the cell membranes, is powered by proton motive force. According to the study, five flagellar proteins, MotA, MotB, FliG, FliM, and FliN, are involved in motor performance. Therefore repression of the *motAB* genes expression in *C. beijerinckii* might be linked to loss of motility during phase transition. Also, the flagellar motility related gene *flgC* is strongly expressed in DSM 6423 during the early exponential phase and down regulated at 6 h at the onset of the stationary phase. Also noticeable is the overexpression of chaperonins *groEL* and *groES* at 8 h. The *groESL* genes are involved in stress response of *Clostridium acetobutylicum* and play a role in solvent tolerance [27]. The toxicity of butanol in particular limits its formation in microbial fermentations. Over-expression of *groESL*, *grpE* and *htpG*, significantly improved butanol tolerance of *C. acetobutylicum* [48].

## Conclusions

*C. beijerinckii* DSM6423 is the most well-known natural isopropanol producer and harbors the secondary alcohol dehydrogenase gene that was cloned in heterologous strains to allow acetone conversion into isopropanol by *C. acetobutylicum* [10, 49, 50]. Improving the natural production of this strain through a targeted approach requires the full sequencing of its genome, together with a transcriptomic analyses. Such analyses were carried out in this study and provide useful data to better understand the genetic background and physiology of this strain.

Notably, this work described a complete genomic study of a natural IBE producer including a first genome physical map of a natural IBE producer. This first analysis highlighted several genetic and metabolic particularities including a specific genomic event occurred on this strain to produce isopropanol. A better understanding of the metabolic pathways and various genes involved opens the door for future targeted approaches to make of this strain an efficient microbial cell factory for isopropanol or IBE production.

## Additional files

Additional file 1: Region of Genome Plasticity (RGP) analysis. (PDF 56 kb)

Additional file 2: Microscopic observation of *C. beijerinckii* DSM6423 cells during glucose fermentation. (PDF 105 kb)

Additional file 3: Details on the 6 biological replicates of *C. beijerinckii* DSM6423 glucose fermentation. (PDF 48 kb)

Additional file 4: List of the most highly transcribed genes during *C. beijerinckii* DSM6423 glucose fermentation at 3 h. (PDF 79 kb)

Additional file 5: Clusters of differential expressed genes in *C. beijerinckii* DSM6423 glucose fermentation. Nine hundred seventy-seven genes differentially expressed at T6 versus T3, or at T8 versus T6 or T11 versus T8 were clustered using CAST algorithm. This table shows the genes included in each cluster, with their ID and their functional annotation, and with their expression using expression at T3 as reference. (PDF 126 kb)

## Abbreviations

ABE: Acetone Butanol Ethanol
HGT: Horizontal Gene Transfert
IBE: Isopropanol Butanol Ethanol
MGE: Mobile Genetic Element
RGPs: Regions of Genomic Plasticity
RPKM: Reads per kilo base per million mapped reads
sADH: Secondary Alcohol Dehydrogenase
