# Correction to: Genome and transcriptome of the natural isopropanol producer Clostridium beijerinckii DSM6423

**DOI:** 10.1186/s12864-018-4799-2

**Published:** 2018-06-01

**Authors:** Hadrien Máté de Gérando, François Wasels, Angélique Bisson, Benjamin Clement, Frédérique Bidard, Etienne Jourdier, Ana María López-Contreras, Nicolas Lopes Ferreira

**Affiliations:** 1 Wageningen Food and Biobased Research, Bornse Weilanden 9, 6709WG Wageningen, The Netherlands; 2 IFP Energies Nouvelles, 1 et 4 avenue de Bois-Préau, 92852 Rueil-Malmaison, France

## Correction

Following the publication of this article [1], the authors noticed that Figs. 2, 3 and 4 were in the incorrect order and thus had incorrect captions. The images that were incorrectly published as Figs. 2, 3 and 4 should have been published as Figs. 3, 4 and 2 respectively.

The correct versions of Figs. 2, 3 and 4 with captions have been included in this Correction.

**Fig. 2:**
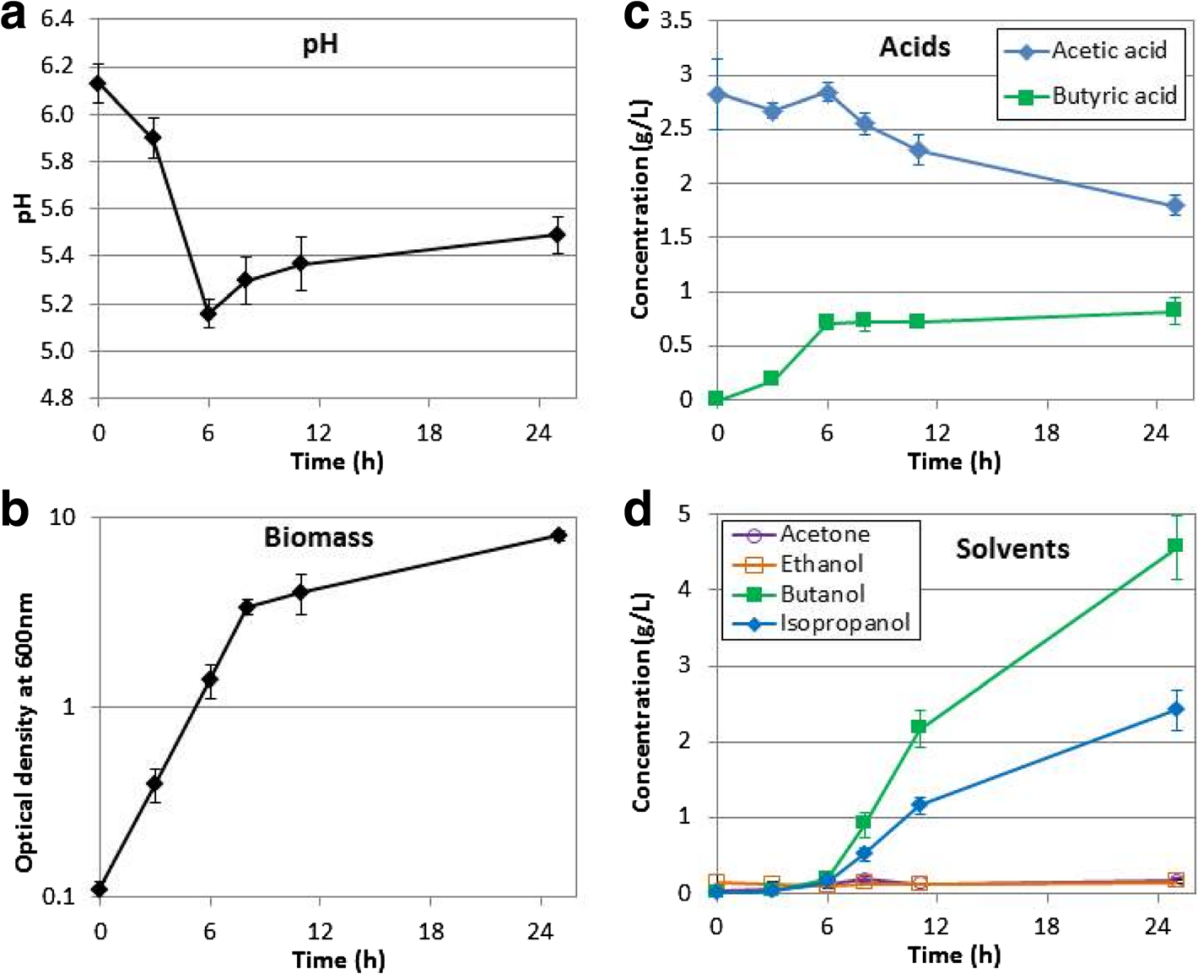
Fermentation profile of *Clostridium beijerinckii* DSM 6423 on glucose. *C. beijerinckii* DSM 6423 was cultivated in bioreactors in GAPES medium. **a** pH, **b** biomass followed by OD_600_, **c** acids and **d** solvents. Values are the mean and standard deviation of the 6 biological replicates. See Additional file 3 for details on the biological replicates

**Fig. 3:**
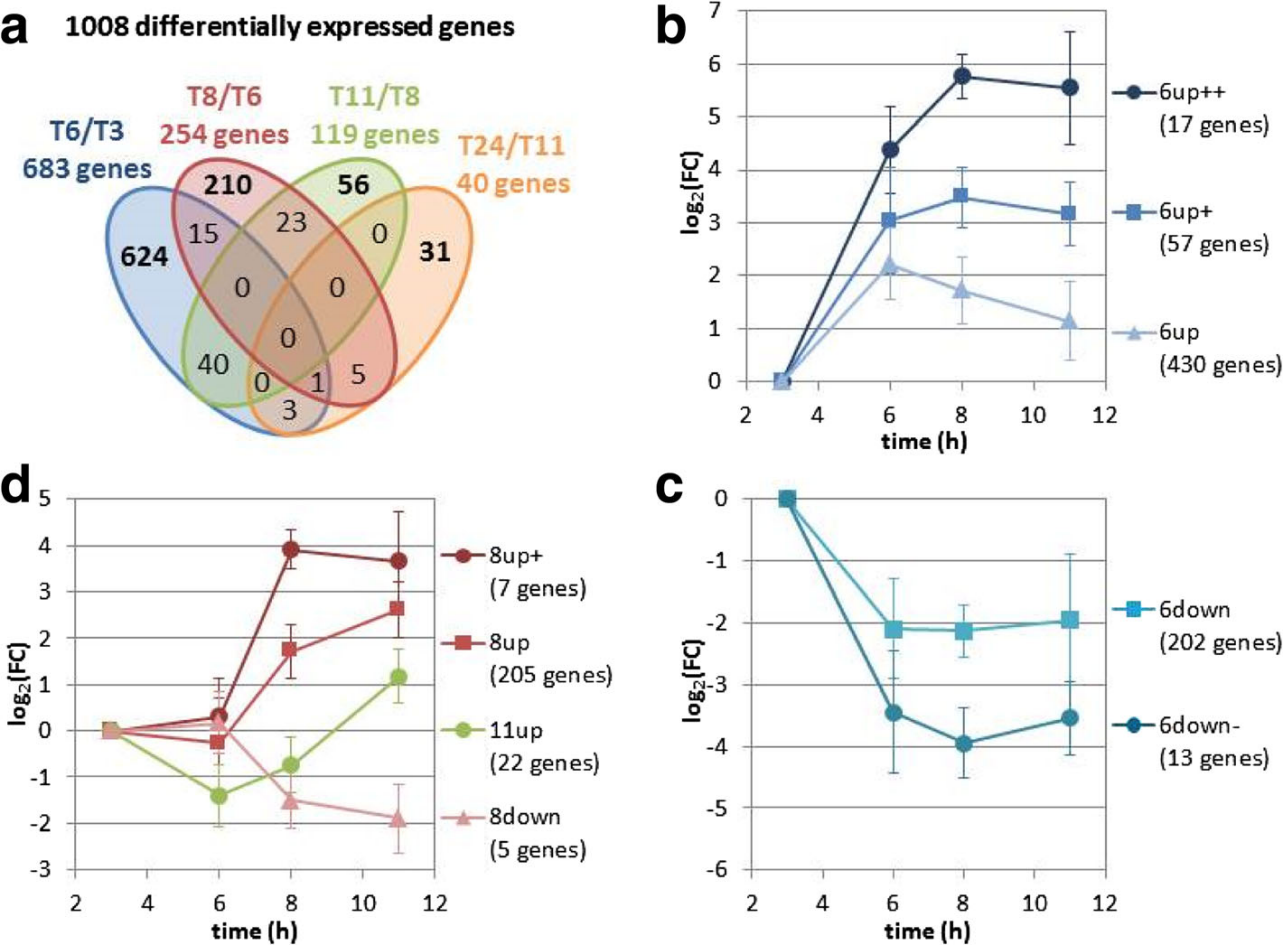
Global transcriptomic analysis of *C. beijerinckii* DSM6423 fermentation on glucose. **a** Venn Diagram showing the number of genes regulated in various physiological time points. **b** to **d**: kinetic expression profiles of various clusters of genes: genes up-regulated at 6 h **b**, genes down-regulated at 6 h **c**, and genes regulated at 8 h or 11 h **d**

**Fig. 4:**
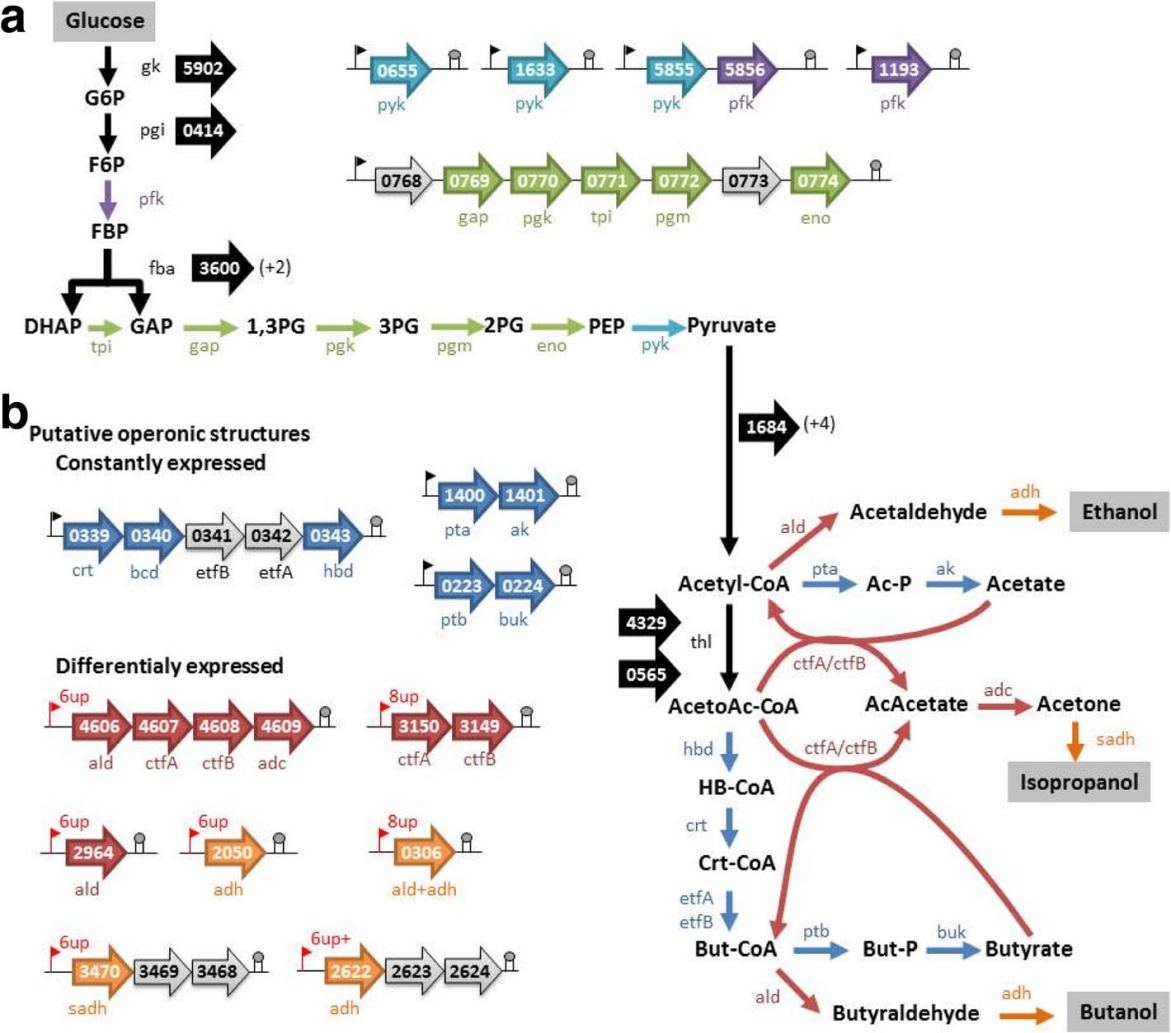
Main genes and predicted operonic structures involved in the central metabolism of in *C. beijerinckii* DSM6423. **a** glycolysis; **b** acids and solvents production) . Number of isozymes, predicted by Microscope tool (Genoscope, Evry, France) are indicated in brackets

The original article has been corrected.
