# Supplementary material for: Genome and transcriptome of the natural isopropanol producer Clostridium beijerinckii DSM6423
Source: BMC Genomics. 2018 Apr 10;19:242. doi: 10.1186/s12864-018-4636-7 (PMC5894183; doi:10.1186/s12864-018-4636-7)
Supplement: Supplementary file 1 — Region of Genome Plasticity (RGP) analysis. (PDF 56 kb) [file 12864_2018_4636_MOESM1_ESM.pdf]

Addition file 1: RGP analysis

Comparison with *Clostridium beijerinckii* NCIMB 8052-chromosome (NC_009617.1)

Label	Begin	End	Length	CLOAB_ NCIMB_8052	Specificity Score
RGP1	326374	340468	14095	73	73
RGP2	377542	398785	21244	100	100
RGP3	401814	416868	15055	91	91
RGP4	445982	498334	52353	96	96
RGP5	854683	867785	13103	100	100
RGP6	1140742	1159053	18312	100	100
RGP7	1350185	1382353	32169	63	63
RGP8	1941408	1952154	10747	86	86
RGP9	2011991	2061467	49477	83	83
RGP10	2281813	2314940	33128	96	96
RGP11	2322667	2329642	6976	100	100
RGP12	2503700	2517596	13897	83	83
RGP13	2737789	2750929	13141	76	76
RGP14	3087490	3095174	7685	100	100
RGP15	3122301	3136140	13840	100	100
RGP16	3140039	3166683	26645	61	61
RGP17	3190549	3196026	5478	40	40
RGP18	3253076	3265677	12602	94	94
RGP19	3322382	3338621	16240	82	82
RGP20	3369230	3389718	20489	93	93
RGP21	3398175	3403762	5588	100	100
RGP22	3463120	3469860	6741	56	56
RGP23	3491558	3511780	20223	100	100
**RGP24**	**3561436**	**3584386**	**22951**	**60**	**60**
RGP25	3611553	3617400	5848	57	57
RGP26	3626925	3632671	5747	45	45
RGP27	3679895	3686442	6548	100	100
RGP28	3778612	3801027	22416	100	100
RGP29	3861023	3870451	9429	75	75
RGP30	3898212	3919804	21593	100	100
RGP31	3936244	3947397	11154	100	100
RGP32	3950518	3974649	24132	89	89
RGP33	4012929	4017944	5016	100	100
RGP34	4022701	4031988	9288	100	100
RGP35	4360559	4371499	10941	73	73
RGP36	4408634	4417861	9228	50	50
RGP37	4454850	4460138	5289	100	100
RGP38	4476930	4503109	26180	65	65
RGP39	4520315	4531672	11358	87	87
RGP40	4660313	4682807	22495	100	100
RGP41	4726188	4755149	28962	69	69
RGP42	5257455	5276600	19146	67	67
RGP43	5375098	5381239	6142	100	100
RGP44	5411127	5428707	17581	77	77
RGP45	5880027	5894342	14316	83	83
RGP46	6030963	6058808	27846	78	78
RGP47	6060983	6076451	15469	93	93

Details of RGP 24

Label	Begin	End	Product	Gene	Codon Adapt. Index	CLOAB_NCIMB 8052
CIBE_v1_3468	3561436	3562830	Sulfide dehydrogenase subunit alpha	sudA	0.836289	+
CIBE_v1_3469	3562830	3563720	conserved protein of unknown function	_	0.773505	+
**CIBE_v1_3470**	**3563746**	**3564801**	**NADP-dependent isopropanol dehydrogenase**	**adh**	**0.641944**	**no correspondance**
CIBE_v1_3471	3565151	3567049	Signal-transduction and transcriptional-control protein	stc	0.641807	+ (weak)
CIBE_v1_3472	3567674	3568948	oxalate decarboxylase	yoaN	0.527902	no correspondance
CIBE_v1_3473	3569560	3571155	NHL repeat containing protein	_	0.645298	no correspondance
CIBE_v1_3474	3571234	3571350	protein of unknown function	_	0.60823	no correspondance
CIBE_v1_3475	3571384	3573222	Histidine kinase	_	0.660303	no correspondance
CIBE_v1_3476	3573364	3573504	conserved protein of unknown function	_	0.690452	no correspondance
CIBE_v1_3477	3573531	3574172	Accessory gene regulator B	_	0.653282	no correspondance
CIBE_v1_3478	3574159	3574626	conserved membrane protein of unknown function	_	0.637122	no correspondance
CIBE_v1_3479	3575061	3577055	Methyl-accepting chemotaxis protein McpA	mcpA	0.679573	+ (weak)
CIBE_v1_3480	3577774	3578430	acetoacetyl CoA-transferase (subunit B)	atoB	0.656718	+
CIBE_v1_3481	3578430	3579086	acetoacetyl CoA-transferase (subunit A)	atoA	0.629413	+ (weak)
CIBE_v1_3482	3579131	3579430	Beta-lactamase	_	0.688745	no correspondance
CIBE_v1_3483	3579435	3580790	ABC transporter substrate-binding protein	_	0.656732	no correspondance
CIBE_v1_3484	3580795	3580938	protein of unknown function	_	0.585267	no correspondance
CIBE_v1_3485	3581189	3582487	Citrate transporter	citN	0.651418	no correspondance
CIBE_v1_3486	3582940	3584283	Fis family transcriptional regulator	_	0.633917	+ (weak)
CIBE_v1_3487	3584267	3584386	protein of unknown function	_	0.588097	no correspondance
CIBE_v1_3488	3584468	3587515	Acriflavin resistance protein	_	0.654431	+
CIBE_v1_3489	3587528	3588790	Hemolysin D	_	0.638424	+
CIBE_v1_3490	3589467	3590165	Transcriptional regulatory protein SrrA	srrA	0.547467	+
CIBE_v1_3491	3590340	3591794	Histidine kinase	_	0.588037	+
