# Supplementary material for: Genome and transcriptome of the natural isopropanol producer Clostridium beijerinckii DSM6423
Source: BMC Genomics. 2018 Apr 10;19:242. doi: 10.1186/s12864-018-4636-7 (PMC5894183; doi:10.1186/s12864-018-4636-7)

Additional file 2

Microscopic observation of *C. beijerinckii* DSM6423 cells during glucose fermentation

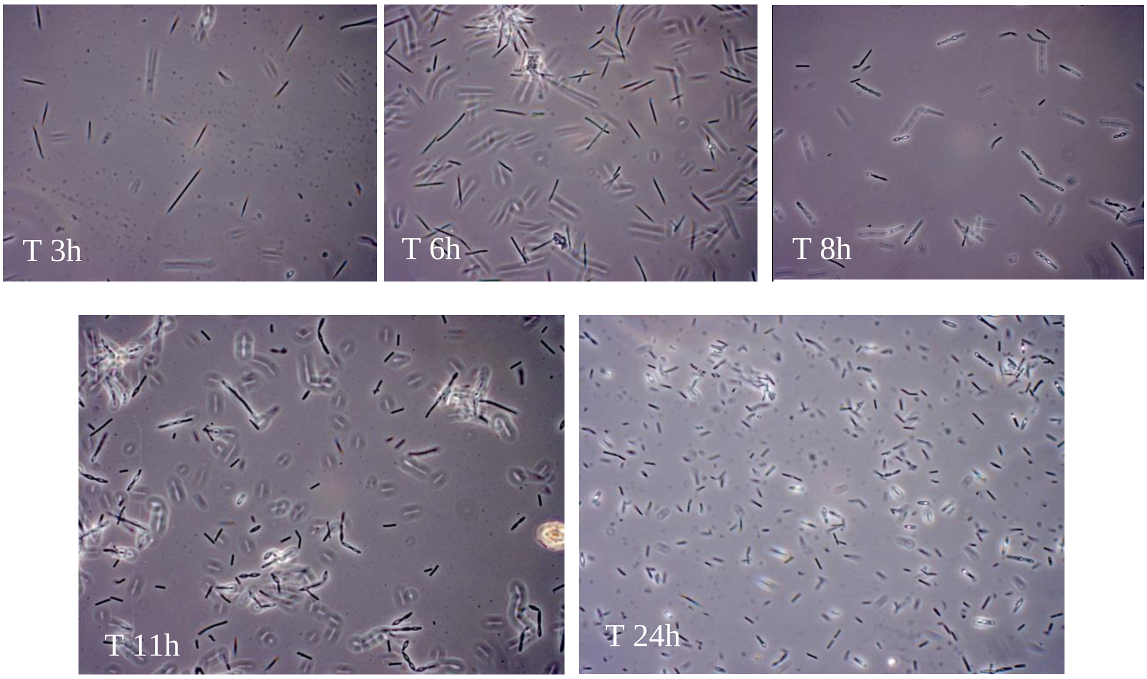

Supplement: Supplementary file 2 — Microscopic observation of C. beijerinckii DSM6423 cells during glucose fermentation. (PDF 105 kb) [file 12864_2018_4636_MOESM2_ESM.pdf]
