# Supplementary material for: Genome and transcriptome of the natural isopropanol producer Clostridium beijerinckii DSM6423
Source: BMC Genomics. 2018 Apr 10;19:242. doi: 10.1186/s12864-018-4636-7 (PMC5894183; doi:10.1186/s12864-018-4636-7)

**Additional file 3**

Details on the 6 biological replicates of *C. beijerinckii* DSM6423 glucose fermentation

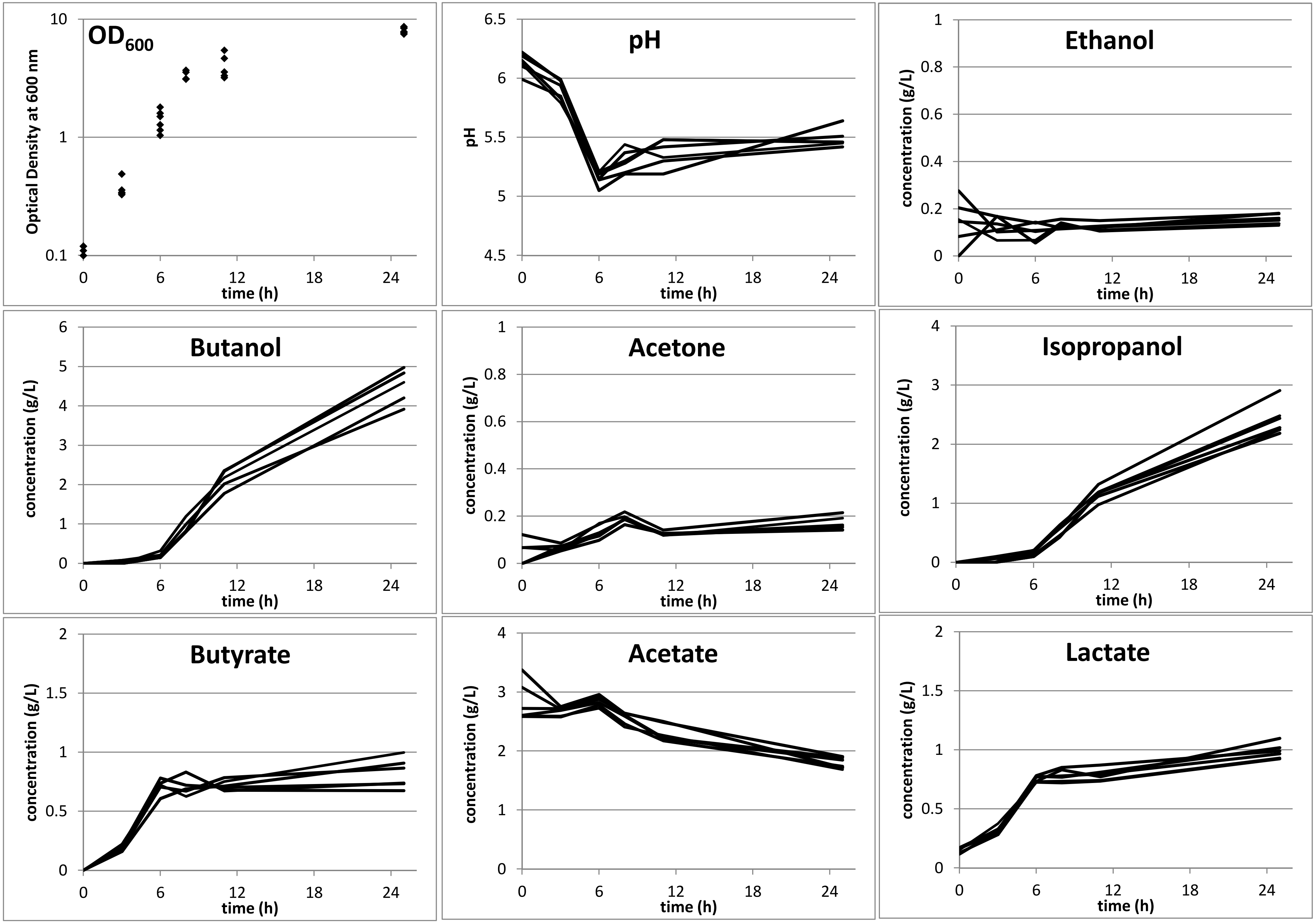

Supplement: Supplementary file 3 — Details on the 6 biological replicates of C. beijerinckii DSM6423 glucose fermentation. (PDF 48 kb) [file 12864_2018_4636_MOESM3_ESM.pdf]
