# Supplementary material for: Genome and transcriptome of the natural isopropanol producer Clostridium beijerinckii DSM6423
Source: BMC Genomics. 2018 Apr 10;19:242. doi: 10.1186/s12864-018-4636-7 (PMC5894183; doi:10.1186/s12864-018-4636-7)
Supplement: Supplementary file 4 — List of the most highly transcribed genes during C. beijerinckii DSM6423 glucose fermentation at 3 h. (PDF 79 kb) [file 12864_2018_4636_MOESM4_ESM.pdf]

Additional file 4: List of the most highly transcribed genes during *C. beijerinckii* DSM6423 glucose fermentation at T3h

Rank	Gene ID	Name	Product	average rpkm
**1**	CIBE_5676	-	putative cell wall binding repeat-containing protein	205.83
**2**	CIBE_5631	-	putative cell wall binding repeat-containing protein	190.15
**3**	CIBE_0314	-	conserved protein of unknown function	188.91
**4**	CIBE_0311	-	conserved protein of unknown function	187.33
**5**	CIBE_0312	-	conserved protein of unknown function	167.41
**6**	CIBE_5303	-	conserved protein of unknown function	131.05
**7**	CIBE_5304	-	conserved protein of unknown function	128.91
**8**	CIBE_0313	-	conserved protein of unknown function	109.01
**9**	CIBE_1020	rpsU	ribosomal protein S21	101.33
**10**	CIBE_6108	ssbA	single-strand DNA-binding protein	99.20
**11**	CIBE_1250	-	Cytochrome C551	97.71
**12**	CIBE_0170	rplB	ribosomal protein L2 (BL2)	97.04
**13**	CIBE_1637	rbr3A	Reverse rubrerythrin-1	86.31
**14**	CIBE_0348	groEL	chaperonin large subunit	84.01
**15**	CIBE_0678	-	Ribonuclease, Rne/Rng family	81.89
**16**	CIBE_4606	eutE	Ethanolamine utilization protein EutE	79.82
**17**	CIBE_6109	rpsF	ribosomal protein S6 (BS9)	77.39
**18**	CIBE_0769	gapA	glyceraldehyde-3-phosphate dehydrogenase	73.96
**19**	CIBE_0169	rplW	ribosomal protein L23	71.38
**20**	CIBE_4608	yodR	putative acyloate-acetoacetate CoA-transferase	71.09
**21**	CIBE_0162	rpsL	ribosomal protein S12 (BS12)	69.39
**22**	CIBE_2172	-	conserved protein of unknown function	68.89
**23**	CIBE_6132	rpmH	ribosomal protein L34	64.12
**24**	CIBE_0061	-	3D domain protein	63.37
**25**	CIBE_5186	nifJ	Pyruvate-flavodoxin oxidoreductase	63.02
**26**	CIBE_3174	rpsU	ribosomal protein S21	62.53
**27**	CIBE_0175	rplP	ribosomal protein L16	61.91
**28**	CIBE_0195	rpsM	ribosomal protein S13	61.36
**29**	CIBE_0168	rplD	ribosomal protein L4	60.49
**30**	CIBE_2171	-	RNA polymerase, sigma 28 subunit, FliA/WhiG family	59.77
**31**	CIBE_0347	groES	chaperonin small subunit	59.31
**32**	CIBE_0161	rpoC	RNA polymerase (beta' subunit)	58.43
**33**	CIBE_0158	rplJ	ribosomal protein L10 (BL5)	57.55
**34**	CIBE_0177	rpsQ	ribosomal protein S17 (BS16)	56.19
**35**	CIBE_5628	-	putative cell wall binding repeat-containing protein	54.39
**36**	CIBE_1402	-	conserved protein of unknown function	51.75
**37**	CIBE_0680	-	conserved protein of unknown function	50.29
**38**	CIBE_4609	adc	Acetoacetate decarboxylase	50.27
**39**	CIBE_0184	rplF	ribosomal protein L6 (BL8)	49.98
**40**	CIBE_0194	-	protein of unknown function	48.34
**41**	CIBE_0178	rplNA	ribosomal protein L14	48.04
**42**	CIBE_0339	yngF	putative Methylglutaconyl-CoA hydratase	47.77
**43**	CIBE_0340	acdA	acyl-CoA dehydrogenase	47.66
**44**	CIBE_0174	rpsC	ribosomal protein S3 (BS3)	46.49
**45**	CIBE_0155	nusG	transcription antitermination factor	46.45
**46**	CIBE_4607	yodS	putative aminoacyloate CoA-transferase	46.44
**47**	CIBE_0183	rpsH	ribosomal protein S8 (BS8)	46.17
**48**	CIBE_2315	fbaA	fructose-1,6-bisphosphate aldolase	45.70
**49**	CIBE_0180	rplE	ribosomal protein L5 (BL6)	44.85
**50**	CIBE_0181	rpsNA	ribosomal protein S14	44.49
**51**	CIBE_0564	mnaA	UDP-N-acetylmannosamine 2-epimerase	44.31
**52**	CIBE_0186	rpsE	ribosomal protein S5	44.07
**53**	CIBE_0173	rplV	ribosomal protein L22 (BL17)	43.64
**54**	CIBE_0100	yabN	putative fusion methylase and nucleotide pyrophosphohydrolase	43.50
**55**	CIBE_0171	-	protein of unknown function	43.38
**56**	CIBE_0167	rplC	ribosomal protein L3 (BL3)	42.93
**57**	CIBE_0166	rpsJ	ribosomal protein S10 (BS13)	42.76
**58**	CIBE_1391	rpmB	ribosomal protein L28	42.55
**59**	CIBE_0133	-	Metal-binding protein of ferredoxin fold	42.54
**60**	CIBE_1413	rpsP	ribosomal protein S16 (BS17)	42.26
**61**	CIBE_1721	-	conserved protein of unknown function	41.91

**62**	CIBE_1305	-	conserved protein of unknown function	41.21
**63**	CIBE_0640	-	conserved protein of unknown function	40.71
**64**	CIBE_0192	-	conserved protein of unknown function	40.56
**65**	CIBE_2132	-	Single-stranded DNA-binding protein	40.26
**66**	CIBE_6107	rpsR	ribosomal protein S18	40.26
**67**	CIBE_6076	-	Acyl-CoA dehydrogenase	40.10
**68**	CIBE_1809	rpmI	ribosomal protein L35	39.31
**69**	CIBE_0172	rpsS	ribosomal protein S19 (BS19)	39.17
**70**	CIBE_0157	rplA	ribosomal protein L1 (BL1)	39.16
**71**	CIBE_0198	rpoA	RNA polymerase (alpha subunit)	38.97
**72**	CIBE_0197	rpsD	30S ribosomal subunit protein S4	38.06
**73**	CIBE_0156	rplK	ribosomal protein L11 (BL11)	37.55
**74**	CIBE_0088	-	protein of unknown function	37.38
**75**	CIBE_0204	rplM	ribosomal protein L13	36.68
**76**	CIBE_0163	rpsG	ribosomal protein S7 (BS7)	36.62
**77**	CIBE_5209	-	conserved exported protein of unknown function	36.30
**78**	CIBE_0203	truA	tRNA pseudouridine synthase A	36.03
**79**	CIBE_0738	-	conserved protein of unknown function	35.47
**80**	CIBE_0187	rpmD	ribosomal protein L30 (BL27)	35.30
**81**	CIBE_0176	rpmC	ribosomal protein L29	35.25
**82**	CIBE_0185	rplR	ribosomal protein L18	34.49
**83**	CIBE_0196	rpsK	ribosomal protein S11 (BS11)	34.31
**84**	CIBE_0179	rplX	ribosomal protein L24 (BL23)	33.84
**85**	CIBE_0191	mapA	methionine aminopeptidase	33.59
**86**	CIBE_0152	tufA	elongation factor Tu	32.91
**87**	CIBE_0153	rpmGA	ribosomal protein L33	32.45
**88**	CIBE_1791	-	conserved protein of unknown function	31.63
**89**	CIBE_0165	tufA	elongation factor Tu	30.63
**90**	CIBE_0154	secE	Protein translocase subunit SecE	30.27
**91**	CIBE_0824	-	Type 11 methyltransferase	30.15
**92**	CIBE_0188	rplO	ribosomal protein L15	29.54
**93**	CIBE_0089	spoVG	regulator required for spore cortex synthesis (stage V sporulation)	28.51
**94**	CIBE_0740	rbr	Rubrerythrin	28.48
**95**	CIBE_5157	hag	flagellin protein	28.42
**96**	CIBE_0182	-	protein of unknown function	28.26
**97**	CIBE_1176	rplY	50S ribosomal protein L25	26.65
**98**	CIBE_0190	adk	adenylate kinase	26.56
**99**	CIBE_0343	mmgB	3-hydroxybutyryl-CoA dehydrogenase	26.51
**100**	CIBE_5882	-	conserved protein of unknown function	26.30
**101**	CIBE_0679	rplU	ribosomal protein L21 (BL20)	26.15
**102**	CIBE_1722	-	conserved protein of unknown function	26.15
**103**	CIBE_1426	rpsB	ribosomal protein S2	26.11
**104**	CIBE_2028	-	conserved protein of unknown function	25.60
**105**	CIBE_6075	-	Beta-lactamase domain protein	24.69
**106**	CIBE_0869	manX	fused mannose-specific PTS enzymes: IIA component ; IIB component	23.91
**107**	CIBE_0870	levF	phosphotransferase system (PTS) fructose-specific enzyme IIC component	23.81
**108**	CIBE_0586	-	conserved protein of unknown function	23.76
**109**	CIBE_0737	prkA	serine protein kinase	23.61
**110**	CIBE_1417	rplS	ribosomal protein L19	23.59
**111**	CIBE_0770	pgk	phosphoglycerate kinase	23.25
**112**	CIBE_1810	rplT	ribosomal protein L20	22.88
**113**	CIBE_1010	dnaK	molecular chaperone	22.70
**114**	CIBE_0341	etfB	Electron transfer flavoprotein subunit beta	22.17
**115**	CIBE_0741	-	conserved protein of unknown function	22.03
**116**	CIBE_0189	secY	Protein translocase subunit SecY	21.81
**117**	CIBE_5055	-	FeoA family protein	21.49
**118**	CIBE_1808	infC	initiation factor IF-3	19.79
**119**	CIBE_0567	atpB	ATP synthase subunit a	19.66
**120**	CIBE_2780	-	conserved protein of unknown function	19.57
**121**	CIBE_0064	-	conserved protein of unknown function	18.51
**122**	CIBE_0105	-	Septum formation initiator	18.44
**123**	CIBE_0739	-	Stage V sporulation protein R	18.06
**124**	CIBE_0572	atpG	ATP synthase gamma chain	17.93
**125**	CIBE_1405	acpA	acyl carrier protein	17.40
**126**	CIBE_0160	rpoB	RNA polymerase (beta subunit)	17.31

**127**	CIBE_4726	-	conserved protein of unknown function	17.24
**128**	CIBE_5187	-	Flavodoxin/nitric oxide synthase	17.21
**129**	CIBE_2018	efp	elongation factor P	17.20
**130**	CIBE_0629	ribU	Riboflavin transporter RibU	17.18
**131**	CIBE_0289	rbr	Rubrerythrin	16.78
**132**	CIBE_2027	-	Stage III sporulation protein AH	16.75
**133**	CIBE_1031	ppdK	Pyruvate, phosphate dikinase	16.64
**134**	CIBE_0571	atpA	ATP synthase (subunit alpha, component F1)	16.63
**135**	CIBE_5226	cysK	cysteine synthase	16.31
**136**	CIBE_1457	ptsH	histidine-containing phosphocarrier protein of the phosphotransferase system (PTS) (HPr protei	16.16
**137**	CIBE_1400	pta	Phosphate acetyltransferase	15.75
**138**	CIBE_0413	sorD	Sorbitol-6-phosphate 2-dehydrogenase	15.74
**139**	CIBE_1414	ylqC	putative RNA binding protein	15.68
**140**	CIBE_1021	yqeY	conserved hypothetical protein	15.68
**141**	CIBE_5894	-	Glycoside hydrolase	15.49
**142**	CIBE_4975	hsp	18 kDa heat shock protein	15.35
**143**	CIBE_0993	spoIIAB	anti-sigma factor (antagonist of sigma(F)) and serine kinase	15.31
**144**	CIBE_3470	adh	NADP-dependent isopropanol dehydrogenase	15.17
**145**	CIBE_5681	-	Cell wall-binding protein	15.11
**146**	CIBE_0536	-	Glycosyl transferase, group 1	15.05
**147**	CIBE_1313	-	conserved exported protein of unknown function	14.94
**148**	CIBE_1427	tsf	elongation factor Ts	14.75
**149**	CIBE_1445	rpsO	ribosomal protein S15 (BS18)	14.64
**150**	CIBE_5968	yaaK	DNA binding protein	14.64
**151**	CIBE_2050	adh	NADPH-dependent butanol dehydrogenase	14.46
**152**	CIBE_4431	-	conserved exported protein of unknown function	14.40
**153**	CIBE_0771	tpiA	triose phosphate isomerase	14.39
**154**	CIBE_1009	grpE	Protein GrpE	14.29
**155**	CIBE_4958	-	conserved protein of unknown function	14.15
**156**	CIBE_1001	rpsT	ribosomal protein S20 (BS20)	14.05
**157**	CIBE_0164	fusA	elongation factor G	14.04
**158**	CIBE_0542	veg	conserved hypothetical protein	13.94
**159**	CIBE_1358	ylmC	essential sporulation protein	13.90
**160**	CIBE_5855	pyk	pyruvate kinase	13.83
**161**	CIBE_0538	yabG	sporulation-specific protease	13.76
**162**	CIBE_1582	clpX	protein unfolding ATPase required for presentation of proteins to proteases	13.74
**163**	CIBE_0224	buk	branched-chain fatty-acid kinase	13.73
**164**	CIBE_5065	glnA	glutamine synthetase	13.69
**165**	CIBE_5054	-	FeoA family protein	13.68
**166**	CIBE_1611	-	conserved exported protein of unknown function	13.66
**167**	CIBE_1586	-	conserved protein of unknown function	13.46
**168**	CIBE_1831	sepF	Cell division protein SepF	13.45
**169**	CIBE_1581	clpP	ATP-dependent Clp protease proteolytic subunit	13.30
**170**	CIBE_0159	rplL	ribosomal protein L12 (BL9)	13.19
**171**	CIBE_0768	-	Transcriptional regulator, DeoR family	13.01
**172**	CIBE_1373	spoIVA	morphogenetic stage IV sporulation protein	13.01
**173**	CIBE_1401	ackA	acetate kinase	12.75
**174**	CIBE_0570	atpH	ATP synthase subunit delta	12.67
**175**	CIBE_5682	-	Cell wall-binding protein	12.59
**176**	CIBE_2783	-	conserved protein of unknown function	12.54
**177**	CIBE_0554	-	Monogalactosyldiacylglycerol synthase	12.51
**178**	CIBE_0065	-	conserved exported protein of unknown function	12.42
**179**	CIBE_5924	glgC	glucose-1-phosphate adenylyltransferase (ADP-glucose pyrophosphorylase) subunit alpha	12.42
**180**	CIBE_1012	dnaJ	co-factor of molecular chaperone	12.10
**181**	CIBE_1403	rpmF	ribosomal protein L32	12.06
**182**	CIBE_0193	infA	initiation factor IF-I	11.96
**183**	CIBE_4106	-	conserved protein of unknown function	11.91
**184**	CIBE_0215	ptsI	phosphotransferase system (PTS) enzyme I	11.81
**185**	CIBE_2041	spo0A	response regulator	11.78
**186**	CIBE_1331	cymR	transcriptional regulator of cysteine biosynthesis	11.69
**187**	CIBE_3665	-	conserved protein of unknown function	11.68
**188**	CIBE_0893	dhaS	3-hydroxypropionaldehyde dehydrogenase	11.62
**189**	CIBE_5170	fliY	flagellar motor switching and energizing phosphatase	11.61
**190**	CIBE_3469	-	conserved protein of unknown function	11.59
**191**	CIBE_2402	yqeA	amino acid (carbamate) kinase	11.58

**192**	CIBE_0342	etfA	electron transfer flavoprotein (alpha subunit)	11.56
**193**	CIBE_1591	-	conserved protein of unknown function	11.49
**194**	CIBE_4056	dfx	Desulfoferrodoxin	11.47
**195**	CIBE_0774	eno	enolase	11.20
**196**	CIBE_0992	spoIIAA	Anti-sigma F factor antagonist	11.20
**197**	CIBE_6021	-	conserved exported protein of unknown function	11.20
**198**	CIBE_0318	-	ABC transporter, ATP-binding protein	11.14
**199**	CIBE_0104	-	Spore cortex biosynthesis protein YabQ	11.09
**200**	CIBE_2139	asd	aspartate-semialdehyde dehydrogenase	11.01
**201**	CIBE_2381	-	protein of unknown function	10.93
**202**	CIBE_5923	-	Glucose-1-phosphate adenylyltransferase	10.88
**203**	CIBE_4919	-	SAM-dependent methyltransferase	10.85
**204**	CIBE_0569	atpF	ATP synthase subunit b	10.67
**205**	CIBE_1455	rny	endoribonuclease Y	10.58
**206**	CIBE_3410	opuCA	glycine betaine/carnitine/choline/choline sulfate ABC transporter (ATP-binding protein)	10.48
**207**	CIBE_2131	-	Deacetylase	10.44
**208**	CIBE_1580	tig	prolyl isomerase (trigger factor)	10.43
**209**	CIBE_2390	-	Sporulation stage II, protein M	10.36
**210**	CIBE_0568	atpE	ATP synthase subunit c	10.32
**211**	CIBE_5172	-	putative CheW protein	10.25
**212**	CIBE_0573	atpD	ATP synthase (subunit beta, component F1)	10.23
**213**	CIBE_0953	trxA	Thioredoxin	10.18
**214**	CIBE_5707	-	conserved protein of unknown function	10.15
**215**	CIBE_0097	-	Peptidylprolyl isomerase	10.04
**216**	CIBE_5881	acpS	holo-acyl carrier protein synthase	9.94
**217**	CIBE_0280	-	conserved exported protein of unknown function	9.91
**218**	CIBE_1446	pnpA	polynucleotide phosphorylase (PNPase)	9.91
**219**	CIBE_5053	-	Ferrous iron transport protein B	9.78
**220**	CIBE_4850	nox	NADH oxidase	9.74
**221**	CIBE_0223	ptb	phosphate butyryl coenzyme A transferase	9.72
**222**	CIBE_0577	-	Sporulation protein	9.65
**223**	CIBE_1306	-	conserved protein of unknown function	9.56
**224**	CIBE_1008	hrcA	Heat-inducible transcription repressor HrcA	9.45
**225**	CIBE_1340	rsmF	pre-16S ribosomal RNA maturation enzyme	9.39
**226**	CIBE_1344	rnjA	ribonuclease J1	9.39
**227**	CIBE_0871	levG	phosphotransferase system (PTS) fructose-specific enzyme IID component	9.29
**228**	CIBE_5717	-	protein of unknown function	9.26
**229**	CIBE_3001	ftnA	Ferritin	9.11
**230**	CIBE_0106	yabR	putative RNA degradation protein; polyribonucleotide nucleotidyltransferase or phosphorylase	9.02
**231**	CIBE_4104	-	Phage-like element pbsx protein XkdM	8.97
**232**	CIBE_4880	-	Pyruvate flavodoxin/ferredoxin oxidoreductase domain protein	8.94
**233**	CIBE_5173	cheY	regulator of chemotaxis and motility	8.92
**234**	CIBE_5742	-	conserved protein of unknown function	8.91
**235**	CIBE_0994	sigF	RNA polymerase sporulation-specific sigma factor (sigma-F)	8.89
**236**	CIBE_3436	-	Electron transfer flavoprotein, alpha/beta-subunit-like protein	8.74
**237**	CIBE_1333	iscU	scaffold protein	8.74
**238**	CIBE_0579	spoIIID	transcriptional regulator	8.68
**239**	CIBE_0566	-	conserved membrane protein of unknown function	8.62
**240**	CIBE_2026	-	Stage III sporulation protein AG	8.61
**241**	CIBE_3435	-	Electron transfer flavoprotein, alpha subunit-like protein	8.57
**242**	CIBE_1339	yrzL	conserved hypothetical protein	8.52
**243**	CIBE_0578	-	Peptidase M23B	8.45
**244**	CIBE_3980	-	conserved protein of unknown function	8.43
**245**	CIBE_5954	-	conserved protein of unknown function	8.34
**246**	CIBE_5683	-	putative cell wall binding repeat-containing protein	8.34
**247**	CIBE_0114	greA	transcription elongation factor	8.26
**248**	CIBE_1638	-	Appr-1-p processing domain protein	8.24
**249**	CIBE_5856	pfkA	6-phosphofructokinase	8.17
**250**	CIBE_4218	acdA	acyl-CoA dehydrogenase	8.13
