# Supplementary material for: Genome and transcriptome of the natural isopropanol producer Clostridium beijerinckii DSM6423
Source: BMC Genomics. 2018 Apr 10;19:242. doi: 10.1186/s12864-018-4636-7 (PMC5894183; doi:10.1186/s12864-018-4636-7)
Supplement: Supplementary file 5 — Clusters of differential expressed genes in C. beijerinckii DSM6423 glucose fermentation. Nine hundred seventy-seven genes differentially expressed at T6 versus T3, or at T8 versus T6 or T11 versus T8 were clustered using CAST algorithm. This table shows the genes included in each cluster, with their ID and their functional annotation, and with their expression using expression at T3 as reference. (PDF 126 kb) [file 12864_2018_4636_MOESM5_ESM.pdf]

Additional file 5: Clusters of differential expressed genes in *C. beijerinckii* DSM6423 glucose fermentation

Clusters	genes	
name	nb	%
6up++	18	44%
6up+	58	21%
6up	431	1%
6down	203	21%
6down-	14	6%
8up+	8	2%
8up	206	0%
8down	6	1%
11up	23	1%
Unclassified	20	2%
TOTAL	987	

Genes	Cluster	log2(FC vs T3h)				functional annotation	
ID	name	T6/T3	T8/T3	T11/T3	T24/T3	gene name	function
CIBE_0313	6up++	5.3	6.2	7.2	5.8	-	conserved protein of unknown function
CIBE_0312	6up++	5.0	6.3	7.4	5.6	-	conserved protein of unknown function
CIBE_0311	6up++	5.0	6.4	7.0	5.3	-	conserved protein of unknown function
CIBE_0314	6up++	4.1	5.9	7.0	5.8	-	conserved protein of unknown function
CIBE_0315	6up++	5.1	5.9	6.1	5.0	-	Radical SAM-family protein
CIBE_0310	6up++	4.7	6.3	5.9	4.6	-	putative ABC transporter ATP-binding protein
CIBE_3477	6up++	5.5	5.6	5.4	4.4	-	Accessory gene regulator B
CIBE_3478	6up++	5.3	5.8	5.0	4.3	-	conserved membrane protein of unknown function
CIBE_0316	6up++	4.5	5.5	5.6	4.2	-	conserved protein of unknown function
CIBE_4430	6up++	4.4	6.4	5.4	3.6	-	putative permease
CIBE_4429	6up++	3.7	5.7	4.8	3.5	-	putative permease
CIBE_0280	6up++	4.5	4.9	4.3	3.9	-	conserved exported protein of unknown function
CIBE_3828	6up++	4.4	5.6	4.4	3.2	-	Glycosyl transferase
CIBE_4431	6up++	2.7	5.4	5.5	3.6	-	conserved exported protein of unknown function
CIBE_4419	6up++	3.7	5.5	4.4	3.1	-	conserved exported protein of unknown function
CIBE_4428	6up++	3.1	5.4	4.7	3.2	glr	Transporter
CIBE_4418	6up++	3.4	5.4	4.4	2.4	-	conserved exported protein of unknown function
CIBE_3476	6up+	4.4	4.6	4.3	3.6	-	conserved protein of unknown function
CIBE_3751	6up+	3.8	5.2	3.8	3.1	-	conserved protein of unknown function
CIBE_3750	6up+	3.9	4.6	3.7	3.4	-	conserved protein of unknown function
CIBE_4789	6up+	3.7	4.3	3.9	3.5	cfg	putative AgrB-like protein
CIBE_0824	6up+	4.8	3.9	3.8	2.7	-	Type 11 methyltransferase
CIBE_3749	6up+	4.1	4.4	3.5	2.8	-	Peptide synthetase
CIBE_5303	6up+	4.6	4.0	3.1	2.7	-	conserved protein of unknown function
CIBE_2622	6up+	2.8	3.7	3.9	4.0	adh	NADPH-dependent butanol dehydrogenase
CIBE_4040	6up+	3.0	3.3	4.1	3.7	-	Aliphatic sulfonates family ABC transporter, periplsmic ligand-binding protein
CIBE_3079	6up+	4.3	3.5	3.3	2.8	panD	aspartate 1-decarboxylase
CIBE_4039	6up+	2.9	3.2	3.9	4.1	tauB	taurine transporter subunit ; ATP-binding component of ABC superfamily
CIBE_3082	6up+	4.2	3.4	3.0	2.9	-	conserved protein of unknown function
CIBE_4610	6up+	2.1	4.1	4.1	3.3	-	Peptidase C1A, papain
CIBE_4417	6up+	3.1	4.7	3.5	2.2	-	conserved membrane protein of unknown function
CIBE_3475	6up+	3.5	3.7	3.4	2.8	-	Histidine kinase
CIBE_4036	6up+	2.7	3.0	3.9	3.7	-	Thioredoxin reductase
CIBE_3355	6up+	3.8	3.0	3.1	3.3	-	RNA polymerase sigma factor SigI
CIBE_3081	6up+	4.1	3.4	2.9	2.8	panB	ketopantoate hydroxymethyltransferase
CIBE_4038	6up+	2.8	2.8	3.8	3.7	-	Type I phosphodiesterase/nucleotide pyrophosphatase
CIBE_2623	6up+	2.6	3.3	3.8	3.3	-	conserved protein of unknown function
CIBE_5304	6up+	4.3	3.6	2.7	2.4	-	conserved protein of unknown function
CIBE_3537	6up+	2.9	4.5	2.5	3.1	-	putative transcriptional regulator
CIBE_5707	6up+	3.0	3.4	3.6	2.9	-	conserved protein of unknown function
CIBE_0782	6up+	2.2	2.8	3.7	4.1	-	Nitrogenase
CIBE_4041	6up+	2.3	3.1	3.8	3.6	tauC	taurine transporter subunit ; membrane component of ABC superfamily
CIBE_2624	6up+	2.2	3.3	3.8	3.5	sudA	Sulfide dehydrogenase subunit alpha
CIBE_3080	6up+	4.0	3.3	3.1	2.3	panC	pantothenate synthetase
CIBE_3353	6up+	3.5	2.7	2.7	3.4	-	Beta-glucanase (fragment)
CIBE_4861	6up+	1.9	3.8	3.2	3.4	-	conserved protein of unknown function
CIBE_3700	6up+	1.2	3.3	3.9	3.9	-	YIEGIA protein
CIBE_3753	6up+	3.1	3.6	2.4	3.1	-	protein of unknown function
CIBE_3354	6up+	3.8	2.9	2.6	2.8	bglA	Beta-glucanase
CIBE_3748	6up+	3.7	3.7	2.7	2.0	-	putative cyclic peptide transporter
CIBE_4416	6up+	3.2	4.3	3.2	1.2	-	conserved membrane protein of unknown function
CIBE_1176	6up+	4.3	3.3	2.6	1.7	rplY	50S ribosomal protein L25
CIBE_4788	6up+	3.2	3.5	2.6	2.4	-	CHAP domain containing protein
CIBE_3357	6up+	4.2	2.8	2.3	2.5	-	Carbohydrate-binding family V/XII
CIBE_2891	6up+	3.7	3.1	2.3	2.6	-	Electron transport complex, RnfABCDGE type, D subunit
CIBE_6013	6up+	4.4	3.5	1.9	1.7	-	conserved protein of unknown function
CIBE_0281	6up+	3.7	3.9	3.3	0.7	-	conserved membrane protein of unknown function
CIBE_4862	6up+	1.8	3.3	3.1	3.3	-	2-hydroxyglutaryl-CoA dehydratase
CIBE_5605	6up+	2.2	2.8	3.5	3.0	-	conserved protein of unknown function
CIBE_2892	6up+	3.8	3.0	2.2	2.3	-	Electron transport complex, RnfABCDGE type, G subunit
CIBE_5208	6up+	3.0	2.9	3.1	2.4	-	Nucleotidyl transferase
CIBE_0725	6up+	2.5	2.9	3.1	2.5	cstA	carbon starvation-induced membrane protein
CIBE_3837	6up+	3.0	3.7	2.1	2.2	yqaJ	putative nuclease; skin element
CIBE_4414	6up+	2.7	3.7	2.8	1.7	-	Cell wall-associated hydrolase-like protein
CIBE_4037	6up+	2.2	2.3	3.5	2.8	-	Thioredoxin domain
CIBE_5606	6up+	2.2	2.7	3.2	2.8	-	Membrane spanning protein
CIBE_3761	6up+	3.7	2.9	2.4	1.4	-	Multidrug transporter MatE
CIBE_3441	6up+	1.6	3.4	3.1	2.4	-	ABC transporter substrate-binding protein
CIBE_2221	6up+	1.1	3.7	3.5	2.0	ytfJ	Uncharacterized spore protein YtfJ
CIBE_2951	6up+	1.4	3.5	2.8	2.3	-	conserved protein of unknown function
CIBE_2222	6up+	1.4	3.5	3.0	1.6	-	conserved membrane protein of unknown function
CIBE_3534	6up+	1.4	3.3	2.3	2.0	-	putative DnaD-like protein, phage replisome organizer

CIBE_4415	6up+	2.4	3.5	2.6	0.5	-	conserved protein of unknown function
CIBE_0309	6up+	1.4	3.2	2.4	1.1	-	protein of unknown function
CIBE_3352	6up	3.7	3.0	2.1	3.2	-	conserved protein of unknown function
CIBE_3401	6up	3.1	2.8	2.2	3.8	-	conserved protein of unknown function
CIBE_3752	6up	2.9	3.4	2.3	3.1	-	protein of unknown function
CIBE_2321	6up	3.6	2.6	2.5	2.7	-	Membrane-like protein
CIBE_2893	6up	3.6	2.9	2.3	2.5	-	Electron transport complex subunit E
CIBE_0460	6up	1.9	1.7	0.0	-0.2	-	conserved protein of unknown function
CIBE_4600	6up	2.6	1.9	1.1	1.5	-	conserved protein of unknown function
CIBE_3469	6up	2.9	2.8	2.6	2.5	-	conserved protein of unknown function
CIBE_2894	6up	3.4	3.0	2.1	2.1	rsxA	putative inner membrane subunit of an electron transport system
CIBE_4602	6up	2.8	1.3	0.9	0.0	-	ThiJ/PfpI domain protein
CIBE_2895	6up	3.5	2.8	2.1	2.2	-	Protein RnfB
CIBE_3350	6up	3.9	2.3	2.0	2.4	-	Beta and gamma crystallin
CIBE_3770	6up	3.2	2.8	2.7	1.8	-	Multi-sensor signal transduction histidine kinase
CIBE_3468	6up	2.6	2.6	2.5	2.7	sudA	Sulfide dehydrogenase subunit alpha
CIBE_3356	6up	3.6	2.2	1.4	3.3	-	Glycoside hydrolase, family 18 (modular protein)
CIBE_2889	6up	3.2	2.7	2.2	2.2	-	Positive regulator of sigma E, RseC/MucC
CIBE_6055	6up	2.7	2.0	1.6	4.0	pdxS	glutamine amidotransferase for pyridoxal phosphate synthesis
CIBE_3769	6up	2.8	2.6	3.0	1.9	-	conserved protein of unknown function
CIBE_4538	6up	2.4	2.2	2.7	2.9	-	RNA polymerase, sigma-24 subunit, ECF subfamily
CIBE_5205	6up	2.9	2.5	2.8	1.9	phnW	2-aminoethylphosphonate--pyruvate transaminase
CIBE_0282	6up	3.0	3.3	2.4	1.5	-	conserved membrane protein of unknown function
CIBE_6021	6up	3.5	2.6	2.1	1.9	-	conserved exported protein of unknown function
CIBE_p0005	6up	3.8	2.5	1.8	2.0	-	putative Peptidase M10A and M12B matrixin and adamalysin
CIBE_3470	6up	2.6	2.4	2.5	2.4	adh	NADP-dependent isopropanol dehydrogenase
CIBE_3827	6up	3.1	3.2	1.7	2.0	-	protein of unknown function
CIBE_5206	6up	2.8	2.5	2.6	2.0	-	Phosphoenolpyruvate decarboxylase
CIBE_3176	6up	2.8	2.5	2.0	2.7	-	Coagulation factor 5/8 type domain protein
CIBE_3835	6up	2.7	3.2	1.8	2.2	-	putative Phage replisome organizer
CIBE_6054	6up	2.5	1.8	1.5	4.0	pdxT	glutamine amidotransferase for pyridoxal phosphate synthesis
CIBE_3351	6up	3.4	2.5	2.0	1.9	-	Chitinase
CIBE_3419	6up	3.5	2.5	2.0	1.7	-	conserved protein of unknown function
CIBE_4603	6up	2.8	1.5	0.8	0.1	-	protein of unknown function
CIBE_5207	6up	2.7	2.4	2.5	1.9	-	Cytidyltransferase-related domain
CIBE_0579	6up	1.7	2.9	2.7	2.3	spoIIID	transcriptional regulator
CIBE_2890	6up	3.1	2.3	2.0	2.1	rnfC	Electron transport complex subunit RnfC
CIBE_0283	6up	2.6	3.1	2.5	1.3	yybJ	putative ATP-binding cassette protein
CIBE_3110	6up	3.2	2.6	0.9	2.9	-	protein of unknown function
CIBE_3194	6up	3.4	2.0	2.0	2.1	-	GCN5-related N-acetyltransferase
CIBE_3909	6up	3.2	2.2	1.9	2.2	-	protein of unknown function
CIBE_5701	6up	2.7	2.3	1.9	2.5	-	Glutamine transport ATP-binding protein GlnQ (fragment)
CIBE_1173	6up	2.5	1.8	2.0	3.0	-	Membrane protein
CIBE_6000	6up	2.6	2.3	2.5	1.9	-	GntR family transcriptional regulator
CIBE_5300	6up	3.2	2.3	1.8	2.0	-	FHA domain containing protein
CIBE_5290	6up	3.9	2.4	1.8	1.1	-	conserved protein of unknown function
CIBE_2455	6up	2.2	3.4	1.7	1.9	-	conserved protein of unknown function
CIBE_2050	6up	3.5	2.5	1.6	1.6	adh	NADPH-dependent butanol dehydrogenase
CIBE_3200	6up	3.5	2.3	1.8	1.5	-	Cytidine deaminase
CIBE_0834	6up	2.7	2.5	1.9	2.0	amyA	Alpha-amylase
CIBE_1313	6up	3.3	2.5	2.1	1.3	-	conserved exported protein of unknown function
CIBE_5299	6up	3.2	2.3	2.0	1.5	-	Protein phosphatase
CIBE_misc_RNA_38	6up	2.8	2.5	1.9	1.8	-	TPP
CIBE_3423	6up	3.0	1.9	1.8	2.3	-	Amino acid adenylation domain protein
CIBE_misc_RNA_6	6up	2.0	2.7	2.2	2.1	-	ydaO-yuaA
CIBE_3421	6up	2.8	1.9	1.9	2.3	-	Long-chain-fatty-acid--(Acyl-carrier-protein) ligase., 6-deoxyerythronolide-B synthase
CIBE_p0006	6up	3.2	2.5	1.7	1.5	-	exported protein of unknown function
CIBE_3169	6up	3.1	2.6	1.6	1.4	ycbN	putative ABC efflux transporter (ATP-binding protein)
CIBE_1314	6up	3.5	2.4	1.3	1.5	-	protein of unknown function
CIBE_6115	6up	1.6	3.2	1.8	2.0	yyaC	conserved hypothetical protein
CIBE_6012	6up	3.5	2.3	1.4	1.3	-	conserved protein of unknown function
CIBE_3109	6up	2.9	2.0	0.9	2.9	-	conserved protein of unknown function
CIBE_5297	6up	3.0	2.0	2.0	1.6	-	Normocyte binding protein 2b
CIBE_3434	6up	2.9	2.1	1.5	2.1	lctP	L-lactate permease
CIBE_5706	6up	2.4	2.5	2.2	1.4	lagD	Lactococcin-G-processing and transport ATP-binding protein LagD
CIBE_5291	6up	3.4	2.0	1.9	1.2	-	conserved protein of unknown function
CIBE_5288	6up	3.4	2.2	1.8	1.2	-	conserved protein of unknown function
CIBE_4321	6up	1.9	2.2	2.3	2.1	-	RNA polymerase subunit sigma-24
CIBE_6020	6up	3.3	2.6	1.7	0.9	-	conserved protein of unknown function
CIBE_3420	6up	3.1	2.1	1.8	1.5	-	FMN-binding protein
CIBE_0019	6up	1.9	3.1	1.8	1.6	-	conserved protein of unknown function
CIBE_4317	6up	2.8	2.1	1.5	2.0	-	conserved protein of unknown function
CIBE_5296	6up	3.2	2.2	1.9	1.2	-	conserved protein of unknown function
CIBE_5286	6up	3.3	2.0	1.6	1.5	-	YD repeat protein (fragment)
CIBE_1337	6up	1.0	2.8	2.4	2.2	-	conserved membrane protein of unknown function
CIBE_4539	6up	2.1	1.6	2.2	2.5	-	conserved protein of unknown function
CIBE_5302	6up	3.2	2.2	1.6	1.4	-	Heat shock protein DnaJ domain protein
CIBE_1004	6up	1.5	3.3	1.2	2.4	-	conserved exported protein of unknown function
CIBE_5294	6up	3.6	2.2	1.8	0.8	-	conserved protein of unknown function
CIBE_6065	6up	3.0	2.3	1.3	1.7	-	protein of unknown function
CIBE_5999	6up	2.5	1.7	2.3	1.8	-	SAM-dependent methyltransferase
CIBE_1287	6up	1.6	1.9	2.0	2.7	-	Glycosyl transferase
CIBE_1294	6up	1.7	2.0	2.1	2.6	-	membrane protein of unknown function
CIBE_3203	6up	3.3	2.0	1.4	1.5	-	Valyl-tRNA synthetase (fragment)
CIBE_3247	6up	2.9	1.8	1.8	1.8	-	Acetolactate synthase
CIBE_6011	6up	3.2	2.3	1.3	1.5	-	conserved protein of unknown function
CIBE_4689	6up	1.7	1.7	1.9	3.0	-	Alcohol dehydrogenase GroES domain protein
CIBE_5301	6up	2.9	2.2	1.6	1.5	-	conserved protein of unknown function
CIBE_1847	6up	2.6	3.3	1.6	0.5	-	putative pilus biogenesis protein
CIBE_2425	6up	2.7	2.2	1.5	1.7	-	putative molybdenum-pterin-binding protein
CIBE_5287	6up	3.1	1.9	1.7	1.4	-	conserved protein of unknown function

CIBE_5705	6up	2.2	2.4	2.2	1.3	-	conserved protein of unknown function
CIBE_3762	6up	3.0	2.0	1.4	1.7	-	Helix-turn-helix-domain containing protein, AraC type
CIBE_3641	6up	2.2	2.3	1.7	1.9	-	conserved protein of unknown function
CIBE_5295	6up	3.3	1.9	1.6	1.1	-	Molecular chaperone
CIBE_3766	6up	2.3	2.1	2.2	1.3	-	conserved protein of unknown function
CIBE_5804	6up	2.0	2.6	1.6	1.8	-	protein of unknown function
CIBE_4320	6up	1.8	2.0	2.1	1.9	-	conserved protein of unknown function
CIBE_4537	6up	2.0	1.6	2.2	2.1	-	conserved membrane protein of unknown function
CIBE_2940	6up	3.1	2.0	1.3	1.4	-	Lactate racemization operon protein LarA
CIBE_0284	6up	2.3	3.0	2.4	0.2	-	conserved protein of unknown function
CIBE_6001	6up	2.4	1.9	2.1	1.5	-	conserved protein of unknown function
CIBE_5042	6up	2.1	1.5	2.1	2.0	-	Sulfate adenylyltransferase
CIBE_4315	6up	2.9	1.8	1.4	1.7	-	conserved protein of unknown function
CIBE_3435	6up	2.9	2.1	1.4	1.4	-	Electron transfer flavoprotein, alpha subunit-like protein
CIBE_4919	6up	3.1	2.5	1.5	0.6	-	SAM-dependent methyltransferase
CIBE_3422	6up	2.6	1.9	1.8	1.5	-	Major facilitator superfamily MFS_1
CIBE_3167	6up	2.7	1.8	1.1	2.1	-	protein of unknown function
CIBE_2391	6up	0.9	2.4	2.5	1.8	xerD	Tyrosine recombinase XerD
CIBE_1297	6up	1.6	2.2	1.9	1.8	-	Polysaccharide biosynthesis protein
CIBE_4604	6up	3.6	2.4	0.9	0.7	-	conserved membrane protein of unknown function
CIBE_0792	6up	1.0	1.1	2.7	2.8	mccB	cystathionine gamma-lyase and homocysteine gamma-lyase for reverse transsulfuration pathway
CIBE_4729	6up	3.0	1.9	1.5	1.1	-	conserved exported protein of unknown function
CIBE_3637	6up	2.8	2.5	0.8	1.3	-	protein of unknown function
CIBE_6002	6up	2.4	1.7	1.8	1.6	-	Membrane protein
CIBE_4690	6up	1.8	1.4	1.7	2.5	-	Aminotransferase class-III
CIBE_5044	6up	2.0	1.4	2.1	2.0	-	Adenylylsulfate reductase subunit beta
CIBE_4730	6up	2.9	1.9	1.6	1.0	-	Transcriptional regulator, MerR family
CIBE_5050	6up	1.8	1.4	2.1	2.1	sbp	sulfate transporter subunit ; periplasmic-binding component of ABC superfamily
CIBE_0829	6up	2.4	2.2	1.6	1.1	-	conserved protein of unknown function
CIBE_1292	6up	1.5	1.5	1.9	2.4	-	Glycosyl transferase family 2
CIBE_4168	6up	2.2	1.8	1.7	1.6	-	4Fe-4S ferredoxin
CIBE_0046	6up	0.6	2.4	2.2	2.1	-	conserved membrane protein of unknown function
CIBE_3248	6up	2.7	1.4	1.8	1.4	-	NADH:flavin oxidoreductase/NADH oxidase
CIBE_4195	6up	2.3	1.9	1.5	1.6	-	conserved protein of unknown function
CIBE_3197	6up	2.6	1.7	1.8	1.2	-	conserved exported protein of unknown function
CIBE_3432	6up	2.8	1.8	1.3	1.3	acdA	acyl-CoA dehydrogenase
CIBE_5043	6up	2.0	1.3	2.0	2.0	cysD	sulfate adenylyltransferase, subunit 2
CIBE_1115	6up	2.7	2.2	1.2	1.1	-	Fucose 4-O-acetylase
CIBE_3343	6up	2.9	1.5	1.2	1.5	-	protein of unknown function
CIBE_0103	6up	0.7	2.5	2.2	1.8	-	YabP family protein
CIBE_3086	6up	2.7	2.0	1.5	0.8	fabG	3-oxoacyl-[acyl-carrier-protein] reductase FabG
CIBE_2160	6up	0.8	2.4	2.5	1.4	-	Cobalamin synthesis protein, P47K
CIBE_3344	6up	2.8	1.9	1.4	1.1	-	protein of unknown function
CIBE_2454	6up	1.9	2.9	1.1	1.3	-	protein of unknown function
CIBE_2805	6up	2.5	1.9	1.7	1.0	-	GCN5-related N-acetyltransferase
CIBE_3747	6up	1.6	2.3	1.9	1.3	grsB	Gramicidin S synthase 2
CIBE_5298	6up	2.5	1.8	1.6	1.2	-	Serine/threonine protein phosphatase
CIBE_3251	6up	3.5	2.1	1.1	0.4	-	conserved exported protein of unknown function
CIBE_4606	6up	3.2	2.7	2.6	2.5	eutE	Ethanolamine utilization protein EutE
CIBE_2390	6up	0.8	2.4	2.3	1.6	-	Sporulation stage II, protein M
CIBE_3198	6up	3.2	2.2	0.1	1.6	-	conserved exported protein of unknown function
CIBE_3433	6up	2.7	1.8	1.3	1.3	-	2-hydroxy-acid oxidase
CIBE_5289	6up	3.3	1.7	1.2	0.8	-	conserved protein of unknown function
CIBE_2942	6up	2.3	2.0	1.4	1.3	-	Transcriptional regulator
CIBE_1296	6up	1.6	1.7	1.7	2.0	-	Glycosyltransferase
CIBE_5292	6up	3.1	1.8	1.5	0.6	-	conserved protein of unknown function
CIBE_1367	6up	1.5	1.8	1.9	1.7	phoU	Phosphate-specific transport system accessory protein PhoU homolog
CIBE_4731	6up	2.8	2.0	1.6	0.5	-	Acetyltransferase
CIBE_3786	6up	2.0	1.9	1.4	1.7	-	Ser/Thr phosphatase family protein
CIBE_1357	6up	1.8	2.2	1.5	1.4	sigG	RNA polymerase sporulation-specific sigma factor (sigma-G)
CIBE_2403	6up	1.6	1.8	2.0	1.6	hyuA	D-stereospecific phenylhydantoinase
CIBE_4820	6up	2.4	2.0	1.6	0.9	pepO	Neutral endopeptidase PepO
CIBE_2751	6up	1.7	1.3	1.7	2.1	yxjH	putative methyl-tetrahydrofolate methyltransferase
CIBE_2420	6up	2.7	1.8	1.3	1.0	-	Molybdopterin binding domain
CIBE_0299	6up	1.8	1.7	1.9	1.4	-	conserved protein of unknown function
CIBE_3910	6up	2.9	1.7	1.2	0.9	-	protein of unknown function
CIBE_1633	6up	0.7	2.2	2.3	1.6	-	Pyruvate kinase
CIBE_3202	6up	2.9	1.5	1.1	1.3	-	Valyl-tRNA synthetase (fragment)
CIBE_p0012	6up	2.8	2.0	0.9	1.0	-	conserved protein of unknown function
CIBE_4173	6up	2.7	1.9	1.1	1.1	-	Abortive infection protein
CIBE_4540	6up	1.8	1.7	2.0	1.3	-	Membrane protein
CIBE_4251	6up	3.0	1.5	0.9	1.3	-	conserved protein of unknown function
CIBE_0481	6up	2.2	2.5	0.9	1.1	-	protein of unknown function
CIBE_3195	6up	2.8	1.5	0.9	1.4	-	Homocysteine S-methyltransferase
CIBE_3788	6up	2.9	1.8	1.1	0.7	-	protein of unknown function
CIBE_3246	6up	2.4	1.5	1.2	1.5	-	Carboxymuconolactone decarboxylase
CIBE_0218	6up	0.4	2.4	2.0	1.8	-	Cell wall hydrolase, SleB
CIBE_2419	6up	2.3	1.7	1.3	1.3	-	LysR family transcriptional regulator
CIBE_4322	6up	2.0	1.6	1.6	1.4	-	conserved exported protein of unknown function
CIBE_1363	6up	1.6	1.6	1.7	1.6	pstS	phosphate ABC transporter (binding lipoprotein)
CIBE_misc_RNA_2	6up	1.5	1.7	1.1	2.2	-	6S
CIBE_misc_RNA_44	6up	1.8	1.9	1.7	1.2	-	Purine
CIBE_4170	6up	2.0	1.5	1.4	1.6	-	conserved protein of unknown function
CIBE_2964	6up	1.7	2.1	1.5	1.2	-	Aldehyde dehydrogenase
CIBE_misc_RNA_11[illegible]	6up	1.5	1.5	1.2	2.3	-	Glycine
CIBE_2418	6up	1.8	1.7	1.9	1.1	-	Molybdopterin dehydrogenase, FAD-binding
CIBE_0799	6up	0.7	2.3	2.1	1.4	-	2', 3'-cyclic nucleotide 2'-phosphodiesterase
CIBE_0476	6up	2.4	2.5	0.8	0.7	-	protein of unknown function
CIBE_3446	6up	1.8	1.9	1.7	1.1	-	conserved protein of unknown function
CIBE_2132	6up	1.6	1.6	1.9	1.3	-	Single-stranded DNA-binding protein
CIBE_0482	6up	2.4	2.5	0.8	0.6	-	protein of unknown function

CIBE_4468	6up	1.9	1.6	1.5	1.4	lacA	Galactoside O-acetyltransferase
CIBE_p0013	6up	2.7	1.5	1.2	0.9	-	conserved protein of unknown function
CIBE_5803	6up	1.7	1.4	1.4	1.8	-	conserved protein of unknown function
CIBE_5305	6up	2.4	1.5	1.4	1.0	-	conserved protein of unknown function
CIBE_4198	6up	2.4	1.7	1.2	0.9	-	Acetyltransferase, GNAT family
CIBE_4323	6up	2.1	1.5	1.4	1.2	-	conserved exported protein of unknown function
CIBE_4174	6up	2.7	1.8	1.1	0.6	-	Methyltransferase type 12
CIBE_4733	6up	3.1	1.9	1.1	0.0	-	Phosphinothricin N-acetyltransferase
CIBE_0471	6up	2.3	2.4	0.7	0.8	-	conserved protein of unknown function
CIBE_0444	6up	2.6	2.1	0.7	0.7	-	conserved protein of unknown function
CIBE_2826	6up	1.8	2.3	1.1	0.8	glnH	glutamine transporter subunit ; periplasmic binding component of ABC superfamily
CIBE_0250	6up	0.4	1.8	2.0	1.9	-	Extracellular solute-binding protein, family 1
CIBE_2017	6up	0.8	2.7	1.4	1.2	-	conserved protein of unknown function
CIBE_3870	6up	2.7	1.7	1.1	0.6	-	Histidine triad (HIT) protein
CIBE_3168	6up	2.8	2.3	0.8	0.2	-	conserved membrane protein of unknown function
CIBE_2878	6up	1.7	1.3	1.5	1.6	-	Phospho-2-dehydro-3-deoxyheptonate aldolase
CIBE_3782	6up	2.7	1.4	1.0	1.0	-	Acetyltransferase, GNAT family (fragment)
CIBE_4171	6up	2.3	1.6	1.1	1.1	-	exported protein of unknown function
CIBE_3324	6up	2.1	1.7	1.1	1.2	-	conserved protein of unknown function
CIBE_3193	6up	2.6	1.2	1.3	0.9	-	conserved protein of unknown function
CIBE_3287	6up	2.5	1.7	1.5	0.3	-	putative cell wall binding repeat-containing protein
CIBE_4466	6up	2.3	1.6	1.4	0.7	-	conserved protein of unknown function
CIBE_0470	6up	2.2	2.5	0.6	0.6	-	conserved protein of unknown function
CIBE_0537	6up	0.6	2.2	1.8	1.4	-	conserved protein of unknown function
CIBE_3640	6up	1.8	1.8	1.1	1.3	-	conserved protein of unknown function
CIBE_2404	6up	1.6	1.6	1.8	0.8	-	YheO domain protein
CIBE_3436	6up	2.5	1.6	0.9	0.9	-	Electron transfer flavoprotein, alpha/beta-subunit-like protein
CIBE_4413	6up	0.6	2.5	1.7	1.2	-	conserved protein of unknown function
CIBE_5285	6up	2.7	1.8	1.1	0.4	-	conserved protein of unknown function
CIBE_1820	6up	2.0	2.1	1.4	0.5	-	conserved protein of unknown function
CIBE_4252	6up	2.7	1.4	0.9	0.9	-	Radical SAM domain protein
CIBE_3471	6up	2.3	1.4	1.3	0.7	stc	Signal-transduction and transcriptional-control protein
CIBE_3899	6up	2.2	1.7	1.1	0.8	-	Glyoxalase/bleomycin resistance protein/dioxygenase
CIBE_3787	6up	2.3	0.8	0.8	1.9	-	protein of unknown function
CIBE_0473	6up	2.6	2.4	0.6	0.2	-	conserved protein of unknown function
CIBE_2417	6up	1.7	1.5	1.6	1.1	ndhS	Nicotinate dehydrogenase small FeS subunit
CIBE_0486	6up	2.4	2.8	0.7	-0.1	-	conserved protein of unknown function
CIBE_3340	6up	2.3	1.3	1.1	1.1	-	Elongation factor G, domain IV
CIBE_3410	6up	2.2	1.3	1.4	0.9	opuCA	glycine betaine/carnitine/choline/choline sulfate ABC transporter (ATP-binding protein)
CIBE_4467	6up	1.9	1.4	1.5	0.9	-	conserved protein of unknown function
CIBE_2655	6up	2.4	1.7	1.4	0.2	-	Flavodoxin
CIBE_0828	6up	2.2	1.9	0.9	0.7	-	putative AgrB-like protein 1
CIBE_3868	6up	2.4	1.4	1.0	1.0	-	conserved protein of unknown function
CIBE_3249	6up	2.2	1.1	1.5	1.0	-	Histidine kinase
CIBE_4250	6up	2.7	1.6	0.9	0.6	-	conserved protein of unknown function
CIBE_6019	6up	2.5	1.6	1.4	0.2	-	Transcriptional regulator, RpiR family
CIBE_4139	6up	2.8	1.8	0.9	0.3	-	Acetyltransferase
CIBE_3347	6up	2.1	1.5	1.0	1.2	-	ATPase AAA (fragment)
CIBE_0943	6up	2.0	0.8	0.8	2.0	-	O-acetylhomoserine aminocarboxypropyltransferase
CIBE_2343	6up	2.3	1.2	1.0	1.2	fldZ	2-enoate reductase FldZ
CIBE_3199	6up	2.7	1.5	0.9	0.6	-	Nicotinamidase-like amidase
CIBE_3773	6up	1.7	1.5	1.6	0.9	-	Accessory gene regulator B
CIBE_1022	6up	0.5	2.0	1.9	1.2	-	conserved protein of unknown function
CIBE_3397	6up	2.3	1.7	0.9	0.6	-	conserved protein of unknown function
CIBE_2171	6up	2.5	2.1	0.4	0.6	-	RNA polymerase, sigma 28 subunit, FliA/WhiG family
CIBE_3034	6up	0.4	2.0	2.0	1.2	-	conserved protein of unknown function
CIBE_1036	6up	0.5	2.1	1.8	1.2	-	Spore coat protein CotS
CIBE_1458	6up	1.2	2.8	0.9	0.8	-	conserved protein of unknown function
CIBE_4773	6up	2.0	1.1	1.4	1.1	-	conserved protein of unknown function
CIBE_5284	6up	2.3	1.4	0.9	0.9	-	Wall-associated protein WapA (fragment)
CIBE_4253	6up	2.4	1.5	0.9	0.8	-	2-dehydropantoate 2-reductase
CIBE_misc_RNA_61	6up	2.3	1.9	0.6	0.8	-	Intron_gpI
CIBE_4444	6up	2.0	1.2	1.2	1.2	-	MarR family transcriptional regulator
CIBE_2825	6up	2.3	2.6	0.6	0.0	-	GntR family transcriptional regulator
CIBE_4197	6up	2.3	1.5	1.0	0.7	-	cAMP-binding protein
CIBE_3789	6up	2.3	1.4	1.1	0.7	-	conserved protein of unknown function
CIBE_3962	6up	2.3	1.5	1.0	0.7	bglF	fused beta-glucoside-specific PTS enzymes: IIA component ; IIB component ; IIC component
CIBE_4196	6up	1.8	1.5	1.0	1.1	-	Short-chain dehydrogenase
CIBE_2591	6up	2.5	2.1	0.5	0.4	-	conserved protein of unknown function
CIBE_0897	6up	2.0	1.5	0.8	1.1	-	Carbohydrate ABC transporter membrane protein 1, CUT1 family
CIBE_0104	6up	0.4	2.2	1.6	1.2	-	Spore cortex biosynthesis protein YabQ
CIBE_4137	6up	2.7	1.6	1.0	0.1	-	conserved protein of unknown function
CIBE_3826	6up	2.9	2.0	0.4	0.0	-	HAD hydrolase, family IA, variant 3
CIBE_4090	6up	1.6	1.5	1.6	0.7	-	Cell wall-binding protein
CIBE_1638	6up	1.7	1.7	1.4	0.5	-	Appr-1-p processing domain protein
CIBE_0464	6up	2.6	2.6	0.4	-0.2	-	conserved protein of unknown function
CIBE_0477	6up	2.2	2.3	0.6	0.3	-	protein of unknown function
CIBE_3902	6up	2.2	1.1	0.6	1.3	-	protein of unknown function
CIBE_3338	6up	1.8	1.3	1.1	1.2	-	Transcription activator, effector binding
CIBE_5293	6up	3.1	1.3	1.2	-0.2	-	conserved membrane protein of unknown function
CIBE_2782	6up	2.2	1.3	1.0	0.7	-	protein of unknown function
CIBE_4735	6up	2.6	1.5	1.2	0.0	-	putative lipoprotein
CIBE_b0020	6up	2.0	1.9	1.0	0.3	-	protein of unknown function
CIBE_4607	6up	2.8	2.2	2.3	2.4	yodS	putative aminoacyloate CoA-transferase
CIBE_3901	6up	2.0	1.6	1.1	0.5	-	putative cell wall binding repeat-containing protein
CIBE_4712	6up	2.0	1.4	0.9	0.9	-	conserved protein of unknown function
CIBE_3911	6up	2.7	1.4	1.0	0.1	-	Putative cell wall binding repeat protein (fragment)
CIBE_5268	6up	2.0	1.2	0.9	1.1	-	conserved protein of unknown function
CIBE_4140	6up	2.7	1.7	0.4	0.4	-	conserved membrane protein of unknown function
CIBE_3871	6up	2.6	1.4	1.2	0.0	-	conserved protein of unknown function
CIBE_4136	6up	2.6	1.4	0.7	0.4	-	Transcriptional regulator, HxlR family

CIBE_0450	6up	2.3	2.0	0.4	0.5	-	conserved protein of unknown function
CIBE_2172	6up	2.2	1.5	0.8	0.6	-	conserved protein of unknown function
CIBE_4143	6up	3.0	2.1	0.7	-0.7	-	ABC transporter related (fragment)
CIBE_3368	6up	1.8	1.4	1.2	0.6	-	putative membrane protein
CIBE_0472	6up	2.1	2.1	0.6	0.3	-	Minor capsid
CIBE_1572	6up	1.7	1.2	0.9	1.2	hisB	imidazoleglycerol-phosphate dehydratase
CIBE_3903	6up	2.4	1.4	0.7	0.5	-	conserved protein of unknown function
CIBE_3639	6up	2.0	1.6	1.3	0.1	-	GCN5-related N-acetyltransferase
CIBE_2421	6up	2.5	1.4	0.7	0.3	moaA	Cyclic pyranopterin monophosphate synthase
CIBE_4316	6up	2.1	0.9	0.8	1.2	-	conserved exported protein of unknown function
CIBE_4608	6up	3.1	2.5	2.4	2.8	yodR	putative acyloate-acetoacetate CoA-transferase
CIBE_0075	6up	1.8	1.1	1.1	1.0	leuA	2-isopropylmalate synthase 2
CIBE_2021	6up	1.6	2.4	0.8	0.2	-	Stage III sporulation protein AB
CIBE_4445	6up	1.7	1.3	1.1	0.9	-	Beta-lactamase
CIBE_5457	6up	2.3	1.2	0.2	1.3	yrbE	putative oxidoreductase
CIBE_3980	6up	2.1	1.9	0.8	0.1	-	conserved protein of unknown function
CIBE_1281	6up	1.9	1.0	0.4	1.6	-	ABC transporter
CIBE_1570	6up	1.5	0.9	1.3	1.2	hisD	histidinol dehydrogenase
CIBE_6076	6up	2.1	1.1	1.3	0.4	-	Acyl-CoA dehydrogenase
CIBE_3245	6up	2.1	1.3	0.1	1.4	-	PAS/PAC sensor signal transduction histidine kinase
CIBE_0438	6up	2.2	2.0	0.4	0.3	-	protein of unknown function
CIBE_0994	6up	2.5	1.5	0.5	0.3	sigF	RNA polymerase sporulation-specific sigma factor (sigma-F)
CIBE_3000	6up	2.0	1.1	0.9	0.7	-	Intermediate filament protein:ATP/GTP-binding site motif A
CIBE_1450	6up	0.2	1.7	1.6	1.3	tepA	protein export-enhancing protease
CIBE_1240	6up	1.7	1.0	1.1	0.9	ilvE	Branched-chain-amino-acid aminotransferase
CIBE_0055	6up	0.4	1.9	1.5	0.9	-	Spore maturation protein
CIBE_4472	6up	2.5	1.5	0.6	0.1	dkgB	2,5-diketo-D-gluconic acid reductase B
CIBE_1794	6up	2.1	2.0	0.4	0.2	-	conserved protein of unknown function
CIBE_3780	6up	2.0	1.3	0.9	0.5	-	membrane protein of unknown function
CIBE_2201	6up	1.7	1.1	0.9	0.9	-	DegV domain-containing protein CA_C1624
CIBE_5267	6up	1.9	1.1	0.7	1.0	-	YD repeat protein
CIBE_0475	6up	2.3	2.3	0.2	-0.1	-	Bacteriophage Gp15 protein
CIBE_0551	6up	1.3	2.3	0.8	0.3	-	conserved protein of unknown function
CIBE_3869	6up	2.2	0.9	0.6	0.9	-	Metal-dependent phosphohydrolase HD sub domain containing protein
CIBE_5423	6up	1.6	1.4	1.1	0.5	argC	N-acetylglutamate gamma-semialdehyde dehydrogenase
CIBE_5270	6up	1.9	1.1	0.8	0.8	-	Pentapeptide repeat protein
CIBE_4183	6up	1.9	1.4	0.7	0.5	-	Ketopantoate reductase ApbA/PanE, N-terminal domain protein
CIBE_3908	6up	2.4	1.6	0.6	-0.2	-	Cyanophycinase
CIBE_0918	6up	2.5	1.8	0.1	0.1	-	ErfK/YbiS/YcfS/YnhG family protein
CIBE_1608	6up	0.2	1.8	1.4	1.1	-	Membrane protein
CIBE_3790	6up	1.9	1.4	1.1	0.0	-	conserved protein of unknown function
CIBE_3396	6up	2.0	1.3	0.8	0.3	-	conserved protein of unknown function
CIBE_0076	6up	2.2	0.9	0.8	0.5	-	Aconitate hydratase
CIBE_3877	6up	2.2	1.1	0.9	0.2	-	conserved protein of unknown function
CIBE_5882	6up	2.1	1.6	0.4	0.3	-	conserved protein of unknown function
CIBE_2932	6up	2.0	1.3	0.5	0.6	-	protein of unknown function
CIBE_3201	6up	2.4	1.0	0.7	0.4	-	conserved protein of unknown function
CIBE_3367	6up	1.8	1.1	1.0	0.5	-	putative cell wall binding protein
CIBE_0479	6up	1.9	2.0	0.4	0.0	-	Phage minor structural protein (modular protein)
CIBE_5271	6up	1.7	1.0	0.9	0.7	-	conserved protein of unknown function
CIBE_p0004	6up	1.7	1.5	0.8	0.3	-	Putative cell wall binding protein (fragment)
CIBE_1609	6up	2.3	1.4	0.4	0.2	-	conserved protein of unknown function
CIBE_0992	6up	2.4	1.3	0.6	0.0	spoIIAA	Anti-sigma F factor antagonist
CIBE_1686	6up	1.9	1.3	0.5	0.6	kdpB	potassium translocating ATPase, subunit B
CIBE_3185	6up	1.6	1.1	1.0	0.6	-	conserved protein of unknown function
CIBE_6075	6up	2.1	1.0	1.0	0.1	-	Beta-lactamase domain protein
CIBE_4279	6up	2.4	0.8	0.5	0.5	-	conserved protein of unknown function
CIBE_4736	6up	2.3	1.6	0.7	-0.3	-	conserved protein of unknown function
CIBE_3236	6up	1.8	1.3	0.6	0.5	-	Alpha/beta hydrolase fold protein
CIBE_3395	6up	2.1	0.9	1.0	0.1	-	N-acetylmuramoyl-L-alanine amidase family 2
CIBE_5280	6up	1.6	0.9	1.2	0.5	ymdB	conserved hypothetical protein
CIBE_4182	6up	2.0	1.3	0.7	0.2	-	DNA mismatch repair protein MutS domain protein
CIBE_0487	6up	2.1	2.1	0.4	-0.4	-	conserved protein of unknown function
CIBE_1611	6up	2.0	1.3	0.4	0.6	-	conserved exported protein of unknown function
CIBE_0457	6up	1.7	1.7	0.4	0.3	-	conserved protein of unknown function
CIBE_5283	6up	2.0	1.4	0.5	0.2	-	putative transcriptional regulatory protein, AsnC family
CIBE_4142	6up	2.5	1.2	0.3	0.0	-	ABC transporter (fragment)
CIBE_1573	6up	1.6	0.9	0.9	0.7	hisH	amidotransferase (glutaminase)
CIBE_5881	6up	1.8	1.3	0.5	0.5	acpS	holo-acyl carrier protein synthase
CIBE_0478	6up	1.9	2.2	0.6	-0.6	-	putative Phage tail component
CIBE_1335	6up	1.6	1.5	0.9	0.0	-	conserved protein of unknown function
CIBE_4135	6up	2.4	1.1	0.5	0.1	-	Phosphatidylethanolamine-binding protein
CIBE_0467	6up	2.1	2.0	0.2	-0.3	-	protein of unknown function
CIBE_4734	6up	2.1	1.2	0.7	0.1	-	conserved membrane protein of unknown function
CIBE_3290	6up	1.7	1.2	0.7	0.4	-	conserved exported protein of unknown function
CIBE_4138	6up	2.3	1.4	0.6	-0.3	-	conserved membrane protein of unknown function
CIBE_1487	6up	2.2	1.0	0.0	0.7	-	conserved protein of unknown function
CIBE_2304	6up	2.0	0.9	0.6	0.5	-	conserved protein of unknown function
CIBE_0289	6up	1.5	1.0	1.0	0.5	rbr	Rubrerythrin
CIBE_2931	6up	2.0	1.5	0.5	0.0	-	conserved protein of unknown function
CIBE_0461	6up	2.0	1.9	0.2	-0.2	-	conserved protein of unknown function
CIBE_4009	6up	1.9	1.7	0.3	0.0	-	Membrane protein
CIBE_5425	6up	1.5	1.3	0.9	0.2	argB	Acetylglutamate kinase
CIBE_3409	6up	1.7	0.9	0.9	0.4	-	ABC transporter permease
CIBE_0468	6up	2.2	2.1	0.0	-0.4	-	Phage coat protein
CIBE_4134	6up	2.2	1.3	0.3	0.1	-	GCN5 family acetyltransferase
CIBE_5458	6up	1.7	1.0	0.5	0.6	iolD	3D-(3,5/4)-trihydroxycyclohexane-1,2-dione hydrolase
CIBE_0445	6up	2.1	1.6	-0.1	0.2	-	Gp49 homologous (fragment)
CIBE_1162	6up	2.2	0.4	0.9	0.4	-	Polysaccharide biosynthesis protein
CIBE_3905	6up	1.8	0.9	0.6	0.6	-	Alpha/beta hydrolase fold
CIBE_1398	6up	0.4	2.1	0.9	0.4	-	Sporulation integral membrane protein YlbJ

CIBE_4367	6up	1.9	1.4	0.6	-0.1	-	DNA mismatch repair protein MutS (fragment)
CIBE_2568	6up	2.1	1.7	0.2	-0.3	-	putative cell wall binding repeat-containing protein
CIBE_0896	6up	2.0	1.4	0.7	-0.4	-	ABC transporter substrate-binding protein
CIBE_3784	6up	2.1	0.9	0.5	0.0	-	protein of unknown function
CIBE_3281	6up	1.9	1.3	0.2	0.1	gldA	glycerol dehydrogenase, NAD
CIBE_3342	6up	1.9	0.7	0.5	0.3	-	conserved protein of unknown function
CIBE_0469	6up	2.0	2.1	-0.1	-0.4	-	conserved protein of unknown function
CIBE_3893	6up	2.0	0.9	0.6	0.0	pspB	putative phosphoserine phosphatase 2
CIBE_4609	6up	3.2	2.4	2.3	2.7	adc	Acetoacetate decarboxylase
CIBE_0993	6up	2.3	1.1	0.3	-0.2	spoIIAB	anti-sigma factor (antagonist of sigma(F)) and serine kinase
CIBE_2043	6up	1.7	1.2	0.5	0.1	-	conserved protein of unknown function
CIBE_0474	6up	2.0	1.8	-0.1	-0.3	-	conserved protein of unknown function
CIBE_3894	6up	2.1	1.2	0.5	-0.4	-	ADP-ribose pyrophosphatase
CIBE_0594	6up	1.6	1.9	0.0	-0.1	-	conserved membrane protein of unknown function
CIBE_0867	6up	1.7	0.7	0.4	0.5	-	Histidine kinase
CIBE_3172	6up	2.2	1.5	0.5	-1.0	-	conserved protein of unknown function
CIBE_0751	6up	1.7	1.0	0.4	0.2	nth	Endonuclease III
CIBE_4366	6up	1.7	1.1	0.2	0.2	-	DNA mismatch repair protein MutS (fragment)
CIBE_3233	6up	1.9	1.1	0.6	-0.4	-	conserved protein of unknown function
CIBE_3811	6up	1.9	0.8	0.4	0.0	-	Fumarylacetoacetate (FAA) hydrolase
CIBE_1605	6up	0.4	2.0	0.7	0.0	-	Sporulation protein SpoVB
CIBE_0462	6up	1.6	1.6	0.2	-0.2	-	putative SPP1 gp7 family phage head morphogenesis protein
CIBE_2040	6up	3.0	0.9	-0.3	-0.6	-	protein of unknown function
CIBE_0416	6up	1.9	1.0	0.2	-0.1	-	Two component regulator three Y domain protein
CIBE_2305	6up	1.8	0.5	0.3	0.3	-	Methyl-accepting chemotaxis protein
CIBE_3892	6up	2.2	0.6	0.2	-0.2	-	conserved protein of unknown function
CIBE_4975	6up	1.8	0.9	0.1	0.1	hsp	18 kDa heat shock protein
CIBE_0277	6up	1.9	0.8	0.8	-0.5	-	Cyclase
CIBE_0357	6up	2.0	0.8	0.0	0.0	-	protein of unknown function
CIBE_4848	6up	2.1	1.1	0.1	-0.5	-	Methyl-accepting chemotaxis sensory transducer
CIBE_2629	6up	1.5	1.2	0.3	-0.2	-	conserved protein of unknown function
CIBE_5615	6up	1.8	0.9	-0.1	0.2	-	Cell wall hydrolase
CIBE_2933	6up	1.8	0.7	0.2	0.1	-	conserved protein of unknown function
CIBE_5880	6up	2.1	1.1	-0.1	-0.3	-	Carbohydrate kinase
CIBE_0488	6up	1.7	1.9	-0.3	-0.7	-	Glycoside hydrolase, family 25
CIBE_3984	6up	1.6	0.7	0.1	0.2	-	conserved protein of unknown function
CIBE_4984	6up	1.6	0.7	0.4	0.0	-	Alpha amylase, catalytic region
CIBE_3783	6up	1.8	0.7	0.3	-0.2	-	conserved protein of unknown function
CIBE_2783	6up	1.7	0.5	0.6	-0.2	-	conserved protein of unknown function
CIBE_1355	6up	1.5	1.6	0.0	-0.5	-	Peptidase U4
CIBE_2168	6up	1.6	0.7	0.3	0.0	-	ATP-dependent DNA helicase RecQ
CIBE_0435	6up	1.6	1.3	0.2	-0.6	-	conserved protein of unknown function
CIBE_0974	6up	1.9	1.0	0.1	-0.6	-	Universal stress protein
CIBE_0466	6up	1.8	1.8	-0.4	-1.0	-	conserved protein of unknown function
CIBE_4280	6up	1.9	0.8	0.3	-0.6	-	Metallo-beta-lactamase superfamily protein
CIBE_4435	6up	1.8	0.9	0.6	-1.0	-	conserved protein of unknown function
CIBE_5924	6up	1.8	0.8	0.0	-0.4	glgC	glucose-1-phosphate adenylyltransferase (ADP-glucose pyrophosphorylase) subunit alpha
CIBE_2041	6up	2.0	0.6	-0.1	-0.3	spo0A	response regulator
CIBE_0456	6up	0.9	1.6	-0.5	0.3	-	conserved protein of unknown function
CIBE_4732	6up	2.1	1.2	0.1	-1.2	lepB	Signal peptidase I
CIBE_0831	6up	1.5	0.6	0.1	0.0	sudA	Sulfide dehydrogenase subunit alpha
CIBE_5056	6up	2.0	0.5	0.2	-0.6	-	Isocitrate dehydrogenase [NADP]
CIBE_4859	6up	1.8	0.7	-0.2	-0.3	-	Cell wall hydrolase/autolysin
CIBE_0088	6up	1.5	0.7	0.1	-0.4	-	protein of unknown function
CIBE_0307	6up	1.8	0.4	0.1	-0.3	-	Two component transcriptional regulator, LytTR family
CIBE_2617	6up	1.9	1.0	0.1	-1.0	-	conserved protein of unknown function
CIBE_3978	6up	1.8	1.1	-0.4	-0.7	-	conserved protein of unknown function
CIBE_5925	6up	1.6	0.5	-0.2	-0.2	-	Alpha-amylase
CIBE_3846	6up	1.6	0.4	0.1	-0.5	thrZ	threonyl-tRNA synthetase
CIBE_5923	6up	1.9	0.7	-0.3	-0.8	-	Glucose-1-phosphate adenylyltransferase
CIBE_0279	6up	2.2	0.3	0.6	-1.8	-	Methyl-accepting chemotaxis sensory transducer
CIBE_0628	6up	1.7	0.4	-0.3	-0.6	-	protein of unknown function
CIBE_0436	6up	1.5	1.1	-0.7	-0.8	-	Excisionase family
CIBE_3001	6up	1.6	0.1	-0.2	-0.4	ftnA	Ferritin
CIBE_0465	6up	1.8	1.5	-0.6	-1.6	-	protein of unknown function
CIBE_2317	6up	1.2	1.2	-0.5	-1.0	-	conserved protein of unknown function
CIBE_3891	6up	2.0	0.3	-0.4	-1.0	-	conserved membrane protein of unknown function
CIBE_0278	6up	1.9	0.0	0.3	-2.5	-	Transcriptional regulator
CIBE_tRNA57	6down	-1.7	-1.4	-0.1	0.4	-	Ala tRNA
CIBE_4931	6down	-1.7	-1.2	-0.4	-0.3	ydaO	Uncharacterized amino acid permease YdaO
CIBE_1058	6down	-1.6	-0.5	-0.9	-0.9	amtB	ammonium transporter
CIBE_misc_RNA_81	6down	-1.6	-1.5	-0.9	-0.4	-	T-box
CIBE_5854	6down	-1.6	-1.2	-0.9	-0.8	-	putative enzyme
CIBE_3636	6down	-2.1	-1.4	-0.4	-0.7	-	putative galactoside ABC transporter
CIBE_tRNA46	6down	-2.2	-1.5	-0.9	0.0	-	Ile tRNA
CIBE_1055	6down	-1.6	-1.3	-0.9	-1.3	-	DNA topoisomerase
CIBE_0267	6down	-1.6	-1.5	-1.2	-0.9	glmS	Glutamine--fructose-6-phosphate aminotransferase [isomerizing]
CIBE_1412	6down	-1.6	-1.2	-1.1	-1.4	ffh	signal recognition particle-like (SRP) GTPase
CIBE_4870	6down	-1.9	-2.7	-0.1	-0.5	-	conserved protein of unknown function
CIBE_5529	6down	-1.7	-1.3	-1.0	-1.4	-	Glycosyl transferase
CIBE_5213	6down	-1.6	-1.6	-1.0	-1.2	-	Sodium:dicarboxylate symporter
CIBE_5723	6down	-1.9	-1.7	-0.9	-0.8	-	conserved exported protein of unknown function
CIBE_5772	6down	-1.8	-1.3	-0.9	-1.4	-	Methyl-accepting chemotaxis sensory transducer
CIBE_2630	6down	-1.7	-1.5	-1.3	-1.2	-	conserved exported protein of unknown function
CIBE_0191	6down	-1.6	-1.4	-1.3	-1.4	mapA	methionine aminopeptidase
CIBE_0637	6down	-1.8	-1.6	-1.1	-1.4	-	2-nitropropane dioxygenase, NPD
CIBE_5709	6down	-1.8	-1.5	-1.0	-1.6	artQ	high affinity arginine ABC transporter (permease)
CIBE_1369	6down	-1.7	-1.7	-1.3	-1.3	-	Fe-S oxidoreductase
CIBE_0805	6down	-2.0	-1.6	-1.2	-1.1	-	conserved protein of unknown function
CIBE_0418	6down	-2.0	-1.3	-1.0	-1.7	ytzG	putative 16S pseudouridylate synthase
CIBE_1014	6down	-1.7	-1.7	-1.3	-1.3	trmB	tRNA (guanine-N(7)-)-methyltransferase

CIBE_1411	6down	-2.0	-1.2	-1.3	-1.6	ylxM	conserved hypothetical protein
CIBE_5007	6down	-1.7	-1.2	-1.0	-2.3	-	conserved protein of unknown function
CIBE_5698	6down	-2.2	-1.4	-1.3	-1.2	-	Cation diffusion facilitator family transporter
CIBE_5863	6down	-1.9	-1.8	-1.1	-1.5	murB	UDP-N-acetylenolpyruvoylglucosamine reductase
CIBE_4421	6down	-2.3	-1.7	-1.7	-0.7	-	transposase (fragment)
CIBE_1001	6down	-1.8	-1.0	-1.3	-2.2	rpsT	ribosomal protein S20 (BS20)
CIBE_0198	6down	-1.5	-1.3	-1.5	-2.1	rpoA	RNA polymerase (alpha subunit)
CIBE_0566	6down	-1.6	-1.8	-1.3	-1.7	-	conserved membrane protein of unknown function
CIBE_1624	6down	-1.7	-1.5	-1.5	-1.8	-	conserved protein of unknown function
CIBE_4992	6down	-1.5	-1.6	-1.6	-1.8	nupQ	permease of ABC guanosine transporter
CIBE_2872	6down	-1.5	-1.6	-1.3	-2.2	-	Ribose-phosphate pyrophosphokinase
CIBE_5607	6down	-1.8	-1.3	-1.5	-2.1	-	Polysaccharide biosynthesis protein
CIBE_0050	6down	-1.7	-1.4	-1.6	-2.0	regA	HTH-type transcriptional regulator RegA
CIBE_1414	6down	-1.6	-1.5	-1.6	-2.0	ylqC	putative RNA binding protein
CIBE_5948	6down	-1.8	-1.9	-1.4	-1.6	-	putative transcriptional regulator, TetR family
CIBE_2478	6down	-1.7	-2.1	-1.6	-1.3	-	conserved protein of unknown function
CIBE_4210	6down	-1.1	-0.3	-2.4	-3.0	-	Extracellular solute-binding protein, family 1
CIBE_5783	6down	-2.0	-1.6	-1.3	-1.8	-	Dehydrogenase
CIBE_2956	6down	-1.8	-1.5	-1.6	-1.8	-	Polar amino acid ABC transporter, inner membrane subunit
CIBE_4864	6down	-2.5	-1.7	-1.3	-1.3	steT	Serine/threonine exchanger SteT
CIBE_1190	6down	-1.9	-1.8	-1.7	-1.3	-	Tetratricopeptide TPR_2 repeat protein
CIBE_6107	6down	-1.5	-1.6	-1.7	-2.1	rpsR	ribosomal protein S18
CIBE_6116	6down	-2.3	-1.5	-1.4	-1.7	-	transposase (fragment)
CIBE_4945	6down	-2.7	-1.9	-1.1	-1.1	ycaM	putative transporter
CIBE_4830	6down	-1.8	-1.5	-1.6	-1.9	-	Signal peptidase I
CIBE_3454	6down	-2.3	-1.8	-1.7	-1.2	-	protein of unknown function
CIBE_1330	6down	-1.7	-1.6	-1.6	-2.0	rarA	DNA-dependent ATPase
CIBE_1490	6down	-1.8	-1.9	-1.3	-1.9	kup	putative potassium transport system protein kup
CIBE_5914	6down	-1.8	-1.3	-2.0	-1.9	-	Peptidase A8
CIBE_5729	6down	-1.7	-1.7	-1.5	-2.1	-	conserved protein of unknown function
CIBE_5126	6down	-1.5	-2.1	-1.6	-1.7	-	Flagellar hook capping protein (fragment)
CIBE_0735	6down	-1.8	-1.6	-1.4	-2.2	-	conserved protein of unknown function
CIBE_1565	6down	-1.9	-1.9	-1.4	-1.9	iscS	Cysteine desulfurase
CIBE_1427	6down	-1.7	-1.4	-1.5	-2.4	tsf	elongation factor Ts
CIBE_6030	6down	-1.5	-1.6	-1.6	-2.3	-	DEAD/DEAH box helicase domain protein
CIBE_3635	6down	-1.8	-1.2	-1.2	-2.9	-	Rubrerythrin
CIBE_misc_RNA_46	6down	-3.1	-2.4	-0.9	-0.7	-	Purine
CIBE_0162	6down	-1.6	-1.8	-1.6	-2.2	rpsL	ribosomal protein S12 (BS12)
CIBE_2562	6down	-1.6	-1.9	-1.8	-2.0	aspS	Aspartate--tRNA ligase
CIBE_0197	6down	-1.8	-1.6	-1.7	-2.2	rpsD	30S ribosomal subunit protein S4
CIBE_0679	6down	-1.8	-1.6	-1.8	-2.2	rplU	ribosomal protein L21 (BL20)
CIBE_5784	6down	-2.2	-1.5	-1.8	-1.8	-	Xylose isomerase domain protein TIM barrel
CIBE_4906	6down	-3.5	-1.9	-0.9	-1.0	-	conserved membrane protein of unknown function
CIBE_1623	6down	-1.9	-1.7	-1.8	-2.0	-	conserved protein of unknown function
CIBE_2076	6down	-1.8	-1.8	-1.6	-2.2	-	Rrf2 family transcriptional regulator
CIBE_misc_RNA_45	6down	-3.2	-3.2	-0.7	-0.4	-	Purine
CIBE_5855	6down	-1.7	-2.0	-1.5	-2.3	pyk	pyruvate kinase
CIBE_0903	6down	-2.1	-2.4	-1.3	-1.7	-	FMN reductase
CIBE_4960	6down	-1.8	-2.1	-1.7	-1.9	ydeP	Uncharacterized HTH-type transcriptional regulator YdeP
CIBE_1566	6down	-1.7	-2.0	-1.4	-2.4	trmI	persulfide ATP pyrophosphatase involved in tRNA modification
CIBE_5118	6down	-1.6	-2.0	-2.0	-2.0	-	Flagellar biosynthetic protein FlhB
CIBE_4098	6down	-1.6	-1.8	-2.5	-1.7	-	conserved protein of unknown function
CIBE_5130	6down	-1.8	-2.2	-1.4	-2.2	-	Flagellar biosynthesis/type III secretory pathway-like protein
CIBE_0024	6down	-2.1	-1.7	-1.5	-2.3	-	Haloacid dehalogenase
CIBE_0680	6down	-1.6	-1.5	-1.9	-2.6	-	conserved protein of unknown function
CIBE_0025	6down	-2.0	-2.0	-1.6	-2.2	ykpA	ABC efflux transporter (ATP-binding protein)
CIBE_6056	6down	-2.5	-1.8	-1.7	-1.8	-	RNA methyltransferase
CIBE_1403	6down	-1.6	-1.9	-1.9	-2.5	rpmF	ribosomal protein L32
CIBE_5945	6down	-1.7	-2.2	-1.6	-2.4	-	Adenosylhomocysteine nucleosidase
CIBE_1265	6down	-1.8	-2.0	-2.0	-2.2	-	conserved exported protein of unknown function
CIBE_5131	6down	-2.0	-2.3	-1.7	-2.0	fliG	flagellar motor switching and energizing component
CIBE_misc_RNA_43	6down	-3.5	-2.6	-1.1	-0.7	-	Purine
CIBE_2029	6down	-1.8	-1.7	-1.8	-2.7	nusB	N utilization substance protein B homolog
CIBE_4120	6down	-2.3	-2.5	-1.5	-1.7	ydeD	putative permease
CIBE_5135	6down	-1.9	-2.2	-1.6	-2.3	-	Flagellar basal body rod protein FlgB
CIBE_tRNA89	6down	-2.9	-1.8	-2.3	-1.2	-	Asp tRNA
CIBE_1068	6down	-2.7	-2.0	-3.0	-0.4	-	conserved protein of unknown function
CIBE_0298	6down	-2.2	-2.0	-2.1	-1.9	-	conserved protein of unknown function
CIBE_1418	6down	-1.9	-2.0	-1.9	-2.4	rbgA	ribosome biogenesis GTPase A
CIBE_0682	6down	-1.8	-1.9	-1.7	-2.9	obgE	ppGpp-binding GTPase involved in cell portioning, DNA repair and ribosome assembly
CIBE_0158	6down	-1.9	-1.8	-1.9	-2.6	rplJ	ribosomal protein L10 (BL5)
CIBE_1419	6down	-2.2	-1.7	-1.9	-2.5	rnhB	ribonuclease HII
CIBE_5117	6down	-1.6	-2.3	-2.0	-2.4	flhA	component of the flagellar export machinery
CIBE_5334	6down	-3.0	-2.6	-2.1	-0.7	rbsA	ribose ABC transporter (ATP-binding protein)
CIBE_5122	6down	-1.6	-2.1	-1.8	-2.9	-	Flagellar basal body-associated protein FliL
CIBE_5132	6down	-2.1	-2.4	-1.9	-2.0	-	Flagellar M-ring protein
CIBE_2477	6down	-2.1	-2.1	-1.9	-2.4	-	Zinc chelation protein SecC
CIBE_4853	6down	-2.0	-2.1	-2.1	-2.3	-	Methyl-accepting chemotaxis protein
CIBE_5144	6down	-1.6	-2.1	-1.8	-3.1	rmlC	dTDP-4-deoxyrhamnose-3,5-epimerase
CIBE_5944	6down	-1.8	-2.2	-2.1	-2.5	tcdA	tRNA threonylcarbamoyladenosine dehydratase
CIBE_2073	6down	-2.1	-2.8	-1.5	-2.3	-	conserved membrane protein of unknown function
CIBE_0074	6down	-1.8	-2.1	-2.0	-2.7	-	Anaerobic ribonucleoside-triphosphate reductase
CIBE_2295	6down	-3.3	-2.5	-1.2	-1.6	yjeM	putative transporter
CIBE_2930	6down	-2.4	-2.1	-1.9	-2.3	-	Zinc chelation protein SecC
CIBE_4105	6down	-1.9	-2.1	-3.0	-1.7	-	Phage-like element pbsx protein XkdK
CIBE_4283	6down	-2.9	-3.0	-2.6	-0.2	-	conserved protein of unknown function
CIBE_1059	6down	-1.8	-2.4	-1.8	-2.8	-	Methyl-accepting chemotaxis protein
CIBE_3993	6down	-1.9	-2.3	-2.1	-2.6	-	Chemotaxis protein
CIBE_4060	6down	-2.4	-2.3	-2.0	-2.1	-	Sigma54 specific transcriptional regulator, Fis family
CIBE_2075	6down	-3.0	-2.4	-1.5	-2.0	-	RNA helicase
CIBE_4104	6down	-1.8	-2.1	-3.1	-1.9	-	Phage-like element pbsx protein XkdM

CIBE_5324	6down	-2.0	-2.2	-2.7	-2.0	-	Radical SAM domain protein
CIBE_0642	6down	-1.8	-2.1	-2.4	-2.6	-	Metallophosphoesterase
CIBE_1639	6down	-2.5	-1.7	-1.9	-2.9	-	conserved protein of unknown function
CIBE_5123	6down	-1.6	-2.1	-2.0	-3.2	ylzI	putative flagellar protein
CIBE_0159	6down	-1.8	-2.0	-2.1	-3.1	rplL	ribosomal protein L12 (BL9)
CIBE_2115	6down	-3.0	-2.5	-2.5	-0.9	-	protein of unknown function
CIBE_misc_RNA_56	6down	-2.3	-2.1	-1.8	-2.8	-	L10_leader
CIBE_0893	6down	-0.4	-2.2	-2.7	-3.9	dhaS	3-hydroxypropionaldehyde dehydrogenase
CIBE_1621	6down	-2.3	-2.3	-1.7	-2.7	-	Metal-dependent hydrolase of the beta-lactamase superfamily
CIBE_5032	6down	-2.2	-2.5	-2.0	-2.4	-	conserved membrane protein of unknown function
CIBE_4234	6down	-2.2	-2.2	-2.8	-1.9	-	FMN-binding protein
CIBE_1172	6down	-2.6	-2.1	-1.9	-2.5	-	Major facilitator superfamily MFS_1
CIBE_5143	6down	-2.2	-2.5	-2.1	-2.4	-	Glycosyltransferase, group 2 family protein
CIBE_tRNA88	6down	-3.3	-2.2	-2.5	-1.2	-	Phe tRNA
CIBE_2192	6down	-0.6	-2.4	-2.9	-3.3	sufB	FeS cluster formation protein
CIBE_0365	6down	-2.2	-2.3	-2.1	-2.6	-	protein of unknown function
CIBE_5154	6down	-2.0	-2.3	-2.0	-2.9	-	Group 2 glycosyl transferase
CIBE_tRNA47	6down	-1.9	-1.9	-1.8	-3.6	-	Asn tRNA
CIBE_1417	6down	-2.1	-2.1	-2.2	-2.9	rplS	ribosomal protein L19
CIBE_3861	6down	-1.7	-1.8	-2.2	-3.6	gltT	proton/sodium-glutamate symport protein
CIBE_5137	6down	-1.9	-2.4	-1.5	-3.5	-	MotA/TolQ/ExbB proton channel
CIBE_0955	6down	-1.5	-2.4	-2.3	-3.2	-	Thioredoxin reductase
CIBE_4548	6down	-2.7	-2.4	-2.2	-2.1	yiaI	putative hydrogenase, 4Fe-4S ferredoxin-type component
CIBE_1212	6down	-1.8	-2.7	-1.9	-3.0	-	conserved protein of unknown function
CIBE_0681	6down	-2.1	-2.0	-2.3	-3.0	rpmA	ribosomal protein L27 (BL24)
CIBE_1127	6down	-2.3	-2.5	-2.5	-2.1	-	Membrane protein
CIBE_0552	6down	-2.4	-2.2	-2.1	-2.8	pyrG	CTP synthetase
CIBE_2480	6down	-2.6	-2.2	-2.3	-2.4	amtB	ammonium transporter
CIBE_5895	6down	-2.1	-2.1	-2.3	-3.0	-	HAD family hydrolase
CIBE_0522	6down	-1.9	-2.3	-2.5	-2.9	-	Sulfatase
CIBE_0832	6down	-2.8	-2.6	-1.9	-2.3	-	Membrane protein
CIBE_0954	6down	-2.0	-2.3	-2.5	-2.9	bsaA	putative bacillithiol peroxidase
CIBE_1130	6down	-2.0	-2.3	-2.1	-3.2	-	Flavin reductase domain protein, FMN-binding
CIBE_4027	6down	-2.3	-2.7	-2.3	-2.4	-	conserved exported protein of unknown function
CIBE_2191	6down	-0.7	-2.6	-3.1	-3.4	sufC	sulfur mobilizing ABC protein, ATPase
CIBE_1211	6down	-2.3	-2.9	-2.2	-2.6	-	conserved exported protein of unknown function
CIBE_3033	6down	-2.1	-1.9	-2.2	-3.7	-	Cof-like hydrolase
CIBE_3372	6down	-2.1	-3.1	-2.7	-2.1	-	SCP-like extracellular protein
CIBE_5104	6down	-2.3	-2.5	-2.0	-3.3	-	Haloacid dehalogenase
CIBE_0081	6down	-2.9	-2.9	-2.1	-2.3	-	HD family phosphohydrolase
CIBE_1090	6down	-1.9	-2.7	-2.7	-2.9	-	Ig-like domain-containing protein,putative cell wall binding protein (fragment)
CIBE_5342	6down	-1.7	-2.6	-2.5	-3.4	-	conserved protein of unknown function
CIBE_5325	6down	-1.9	-2.3	-3.3	-2.7	-	conserved protein of unknown function
CIBE_5134	6down	-2.3	-2.8	-2.0	-3.1	flgC	flagellar component of cell-proximal portion of basal-body rod
CIBE_0141	6down	-2.0	-2.1	-2.9	-3.3	-	Uncharacterized glutaredoxin-like 8.6 kDa protein in rubredoxin operon
CIBE_1803	6down	-2.6	-2.7	-2.3	-2.8	yfmM	putative ATP-binding protein
CIBE_0288	6down	-2.4	-2.6	-2.3	-3.1	-	Methyl-accepting chemotaxis sensory transducer
CIBE_4949	6down	-2.3	-2.6	-2.5	-2.9	-	Secretion protein HlyD family protein
CIBE_0541	6down	-1.8	-2.2	-3.4	-3.0	-	protein of unknown function
CIBE_5155	6down	-2.3	-2.8	-1.9	-3.4	-	conserved protein of unknown function
CIBE_5138	6down	-2.1	-2.7	-2.2	-3.5	-	Flagellin
CIBE_4324	6down	-2.3	-2.0	-2.7	-3.7	-	Methyl-accepting chemotaxis protein
CIBE_tRNA19	6down	-3.1	-2.7	-2.2	-2.7	-	Asn tRNA
CIBE_5156	6down	-2.3	-2.5	-2.2	-3.8	-	Glycosyltransferase
CIBE_5136	6down	-2.8	-2.6	-1.4	-3.9	-	OmpA/MotB domain protein
CIBE_5091	6down	-2.9	-2.6	-2.0	-3.3	hpt	Hypoxanthine phosphoribosyltransferase
CIBE_5694	6down	-2.5	-2.6	-3.7	-2.0	-	exported protein of unknown function
CIBE_5133	6down	-2.2	-2.4	-2.0	-4.0	fliE	Flagellar hook-basal body complex protein FliE
CIBE_5946	6down	-2.2	-2.8	-2.5	-3.4	-	Methyl-accepting chemotaxis sensory transducer
CIBE_5061	6down	-2.4	-2.3	-2.9	-3.2	-	putative Methyl-accepting chemotaxis protein
CIBE_0351	6down	-3.2	-3.6	-2.0	-2.2	guaA	GMP synthetase
CIBE_0539	6down	-1.6	-2.5	-3.1	-3.8	gpo	Glutathione peroxidase
CIBE_5693	6down	-2.3	-2.8	-2.8	-3.1	-	S-layer domain-containing protein (fragment)
CIBE_4777	6down	-2.4	-2.8	-2.6	-3.2	-	Methyl-accepting chemotaxis protein
CIBE_4855	6down	-2.3	-2.3	-3.2	-3.3	-	Chemotaxis protein CheA
CIBE_3862	6down	-1.7	-2.4	-3.5	-3.6	ansB	L-aspartase (aspartate ammonia lyase)
CIBE_1125	6down	-2.2	-3.1	-2.5	-3.5	-	Murein hydrolase transporter LrgA
CIBE_4550	6down	-2.8	-3.1	-2.9	-2.5	-	Molybdopterin-guanine dinucleotide biosynthesis protein B
CIBE_5335	6down	-2.6	-2.2	-3.6	-2.8	mglB	methyl-galactoside transporter subunit ; periplasmic-binding component of ABC superfamily
CIBE_4284	6down	-2.4	-3.1	-3.8	-2.0	-	ApbE family lipoprotein
CIBE_1062	6down	-2.8	-2.8	-2.4	-3.5	-	Methyl-accepting chemotaxis protein
CIBE_2186	6down	-3.2	-3.3	-2.4	-2.7	fruK	fructose-1-phosphate kinase
CIBE_3458	6down	-3.4	-2.7	-3.0	-2.5	-	Methyl-accepting chemotaxis protein
CIBE_4553	6down	-3.0	-3.2	-3.0	-2.5	-	conserved protein of unknown function
CIBE_1126	6down	-2.2	-3.7	-2.6	-3.3	-	protein of unknown function
CIBE_2185	6down	-2.8	-3.3	-2.5	-3.3	fruR	transcriptional regulator (DeoR family)
CIBE_4551	6down	-3.3	-3.4	-2.6	-2.6	-	Molybdenum cofactor biosynthesis protein
CIBE_2187	6down	-3.0	-3.5	-2.6	-2.9	fruA	phosphotransferase system (PTS) fructose-specific enzyme IIABC component
CIBE_3631	6down	-1.6	-2.6	-3.1	-4.6	-	Cation transporter
CIBE_5785	6down	-2.0	-3.0	-3.2	-3.8	-	Oxidoreductase
CIBE_4844	6down	-3.2	-3.0	-2.7	-3.3	maeB	NADP-dependent malic enzyme (conversion of malate into pyruvate)
CIBE_4856	6down	-2.8	-3.1	-3.4	-3.4	cheY	regulator of chemotaxis and motility
CIBE_0214	6down	-2.6	-2.6	-3.0	-4.6	nrdB	Ribonucleoside-diphosphate reductase subunit beta
CIBE_4282	6down	-2.0	-3.7	-3.4	-4.0	-	FMN-binding domain protein
CIBE_0213	6down	-2.5	-3.3	-3.0	-4.5	-	protein of unknown function
CIBE_3630	6down	-2.7	-3.4	-3.5	-3.9	csoR	repressor of copper utilisation proteins - Cu(I)
CIBE_1061	6down	-3.0	-3.4	-2.6	-4.5	-	Transferase
CIBE_0212	6down	-3.0	-3.2	-3.5	-4.6	nrdA	Ribonucleoside-diphosphate reductase subunit alpha
CIBE_2867	6down-	-3.8	-4.0	-1.9	-2.2	-	conserved membrane protein of unknown function
CIBE_4061	6down-	-4.1	-3.5	-2.9	-2.1	-	Glycoside hydrolase, family 18
CIBE_4547	6down-	-2.3	-4.2	-3.2	-3.4	hndD	NADP-reducing hydrogenase subunit HndC

CIBE_4549	6down-	-2.8	-4.5	-3.0	-2.8	fdhD	Protein FdhD homolog
CIBE_2868	6down-	-4.1	-4.5	-2.0	-2.7	xpt	xanthine phosphoribosyltransferase
CIBE_4976	6down-	-2.4	-4.4	-3.3	-5.1	-	Methyl-accepting chemotaxis sensory transducer
CIBE_4552	6down-	-3.5	-3.6	-3.9	-4.5	fdhF	Formate dehydrogenase H
CIBE_4062	6down-	-3.1	-3.6	-4.3	-4.8	ytbE	promiscuous aldo/keto reductase
CIBE_0397	6down-	-3.4	-4.1	-3.9	-4.5	-	Bacterial surface protein
CIBE_1759	6down-	-3.5	-3.5	-4.5	-5.0	gltT	proton/sodium-glutamate symport protein
CIBE_1119	6down-	-3.8	-3.6	-4.2	-5.0	-	conserved membrane protein of unknown function
CIBE_4063	6down-	-3.1	-4.0	-4.6	-5.4	-	FMN reductase
CIBE_1120	6down-	-4.8	-3.8	-4.5	-6.4	-	conserved membrane protein of unknown function
CIBE_3701	8up+	1.1	3.7	4.0	3.9	-	conserved protein of unknown function
CIBE_5020	8up+	0.3	5.1	3.9	3.1	bceA	bacitracin ABC efflux transporter (ATP-binding protein)
CIBE_1732	8up+	0.4	2.8	4.6	3.9	yraG	putative spore coat protein
CIBE_4645	8up+	0.5	4.7	3.2	3.1	-	Erythromycin esterase
CIBE_3257	8up+	-0.3	2.8	4.6	3.8	-	PTS beta-glucoside transporter subunit IIBC
CIBE_4643	8up+	0.3	3.9	2.6	2.8	yxdL	ABC transporter (ATP-binding protein); efflux of cationic peptides
CIBE_5019	8up+	-0.2	4.4	2.8	2.6	-	conserved membrane protein of unknown function
CIBE_0318	8up	0.5	3.1	4.1	4.5	-	ABC transporter, ATP-binding protein
CIBE_2546	8up	0.3	2.4	2.8	6.4	-	protein of unknown function
CIBE_0317	8up	0.4	3.1	3.9	4.5	-	ABC transporter, permease protein
CIBE_5103	8up	0.6	3.1	3.8	4.2	-	Membrane protein
CIBE_3697	8up	0.1	2.4	2.1	6.9	-	conserved protein of unknown function
CIBE_5102	8up	0.8	2.8	3.6	4.0	-	Diguanylate cyclase
CIBE_1783	8up	-0.1	1.5	4.6	5.1	-	conserved protein of unknown function
CIBE_2954	8up	0.5	2.4	3.7	3.9	-	conserved protein of unknown function
CIBE_4796	8up	-0.4	2.9	4.4	3.6	-	conserved protein of unknown function
CIBE_3575	8up	-0.3	2.8	4.4	3.5	cooS	Carbon monoxide dehydrogenase
CIBE_4568	8up	0.7	2.9	3.5	3.3	yvcR	putative ABC transporter (ATP-binding protein)
CIBE_0820	8up	0.1	2.2	4.2	3.6	-	Polysaccharide deacetylase
CIBE_5655	8up	0.3	2.8	3.6	3.5	-	Tyrosine protein kinase
CIBE_2308	8up	0.2	2.5	3.9	3.4	-	LemA family protein
CIBE_1684	8up	-0.5	2.1	4.2	3.8	nifJ	Pyruvate-flavodoxin oxidoreductase
CIBE_3300	8up	0.6	2.8	3.6	2.5	cspC	cold-shock protein
CIBE_1784	8up	-0.5	1.2	4.0	4.7	rpsD	30S ribosomal protein S4 B
CIBE_3596	8up	0.2	2.4	3.9	2.9	-	Alkyl hydroperoxide reductase/ Thiol specific antioxidant/ Mal allergen
CIBE_1336	8up	1.2	3.0	2.8	2.3	-	PRC-barrel domain protein
CIBE_1733	8up	0.0	2.2	3.8	3.2	yraF	putative spore coat protein
CIBE_5645	8up	0.3	2.9	3.1	3.0	-	Glycosyl transferase, group 1
CIBE_3725	8up	0.2	2.4	3.4	3.3	-	D-galactose-binding periplasmic protein
CIBE_5519	8up	-0.3	2.4	4.2	2.9	-	conserved protein of unknown function
CIBE_3507	8up	0.7	2.2	3.4	2.9	-	conserved protein of unknown function
CIBE_3163	8up	0.4	2.0	3.4	3.4	celB	Cellobiose permease IIC component
CIBE_0545	8up	0.4	2.6	3.2	3.0	-	Cyanophycinase
CIBE_2771	8up	1.3	2.8	2.9	2.1	-	conserved protein of unknown function
CIBE_2045	8up	-0.1	2.9	3.4	2.7	-	conserved protein of unknown function
CIBE_3260	8up	-1.2	2.3	4.1	3.6	-	Transaldolase
CIBE_5520	8up	-0.5	2.1	4.3	3.0	-	Coat F domain protein
CIBE_1086	8up	-0.3	3.0	3.2	2.8	-	conserved exported protein of unknown function
CIBE_5517	8up	-0.6	2.5	4.2	2.6	-	conserved protein of unknown function
CIBE_5328	8up	0.0	2.0	3.0	3.6	-	putative galactoside ABC transporter
CIBE_3572	8up	0.0	2.1	3.5	3.0	norV	flavorubredoxin oxidoreductase
CIBE_0127	8up	0.9	2.6	2.8	2.2	-	Amidohydrolase
CIBE_0098	8up	-0.2	2.9	3.5	2.4	spoVT	transcriptional regulator
CIBE_2663	8up	-0.8	2.6	3.6	3.1	-	Amidohydrolase
CIBE_0544	8up	0.1	2.8	2.8	2.5	-	conserved protein of unknown function
CIBE_3528	8up	0.1	2.5	3.5	2.2	-	Coat F domain protein
CIBE_3577	8up	-0.2	1.4	3.6	3.5	-	Cupin 2 conserved barrel protein
CIBE_0546	8up	0.2	2.2	3.1	2.9	-	Cyanophycin synthetase
CIBE_5454	8up	0.6	2.1	3.2	2.3	yngF	putative Methylglutaconyl-CoA hydratase
CIBE_2057	8up	0.0	1.9	3.0	3.3	-	Glycosyl transferase
CIBE_3642	8up	0.6	2.5	2.4	2.7	-	D-galactose-binding periplasmic protein
CIBE_3958	8up	0.6	2.5	3.1	2.0	-	conserved protein of unknown function
CIBE_5420	8up	-1.2	2.2	3.6	3.6	-	conserved protein of unknown function
CIBE_4858	8up	0.3	2.1	3.1	2.7	bglB	Thermostable beta-glucosidase B
CIBE_2362	8up	-0.3	2.0	3.3	3.1	yhcV	CBS domain-containing protein YhcV
CIBE_1750	8up	0.4	1.5	3.3	2.8	-	conserved protein of unknown function
CIBE_2002	8up	0.6	2.2	2.9	2.2	-	conserved protein of unknown function
CIBE_4218	8up	0.8	2.4	2.7	2.1	acdA	acyl-CoA dehydrogenase
CIBE_4985	8up	-0.2	2.8	3.2	2.2	-	conserved exported protein of unknown function
CIBE_4745	8up	-0.6	2.0	3.2	3.4	-	putative galactoside ABC transporter
CIBE_1188	8up	-0.7	1.2	3.8	3.7	-	Delta-lactam-biosynthetic de-N-acetylase
CIBE_5101	8up	-0.3	2.3	3.2	2.7	-	conserved protein of unknown function
CIBE_4567	8up	0.4	2.1	2.5	2.8	-	conserved membrane protein of unknown function
CIBE_0757	8up	0.6	2.2	3.0	2.1	-	conserved protein of unknown function
CIBE_1745	8up	-0.9	1.5	4.1	3.1	-	conserved protein of unknown function
CIBE_1226	8up	0.4	2.1	2.8	2.4	-	conserved protein of unknown function
CIBE_0065	8up	0.4	2.3	2.7	2.3	-	conserved exported protein of unknown function
CIBE_1734	8up	-0.3	1.5	3.4	2.9	adhB	putative oxidoreductase
CIBE_3627	8up	-0.2	1.9	3.2	2.7	-	FAD-dependent oxidoreductase
CIBE_1669	8up	-0.3	2.1	3.1	2.6	amyC	putative starch degradation products transport system permease protein AmyC
CIBE_5518	8up	-0.6	1.9	3.7	2.5	-	Coat F domain protein
CIBE_3927	8up	-0.1	2.6	2.2	2.8	-	conserved protein of unknown function
CIBE_0306	8up	-0.7	1.9	3.6	2.6	adhE	fused acetaldehyde-CoA dehydrogenase ; iron-dependent alcohol dehydrogenase ; pyruvate-formate lyase deactivase
CIBE_1229	8up	-0.8	2.3	3.1	2.8	-	Stage V sporulation protein AF
CIBE_4835	8up	0.2	1.7	3.0	2.5	-	Ferredoxin hydrogenase
CIBE_2743	8up	-0.4	2.3	3.2	2.2	-	conserved protein of unknown function
CIBE_1494	8up	-0.2	1.3	3.2	2.9	-	Protein CA_C1420
CIBE_5646	8up	0.3	2.3	2.7	1.9	-	conserved membrane protein of unknown function
CIBE_2218	8up	-0.9	1.6	3.6	3.0	dacF	D-alanyl-D-alanine carboxypeptidase DacF
CIBE_2307	8up	-0.5	1.5	3.1	3.1	-	conserved exported protein of unknown function
CIBE_5419	8up	-1.0	1.7	2.9	3.6	-	conserved protein of unknown function

CIBE_2131	8up	0.4	2.0	2.6	2.2	-	Deacetylase
CIBE_2056	8up	-0.2	1.9	3.2	2.2	-	conserved protein of unknown function
CIBE_3019	8up	-0.9	2.2	3.6	2.2	-	conserved protein of unknown function
CIBE_3504	8up	-1.1	0.8	3.4	4.0	-	conserved exported protein of unknown function
CIBE_1735	8up	-0.1	1.9	2.8	2.5	-	Spore coat protein
CIBE_2566	8up	0.3	2.0	2.8	2.0	-	conserved protein of unknown function
CIBE_1545	8up	0.3	1.8	2.8	2.2	-	conserved protein of unknown function
CIBE_1683	8up	0.2	2.2	2.7	2.0	-	conserved protein of unknown function
CIBE_3923	8up	0.1	1.6	2.7	2.6	-	Spore cortex-lytic protein
CIBE_1309	8up	-0.8	2.4	2.4	2.9	-	protein of unknown function
CIBE_5365	8up	-0.9	0.8	3.2	3.7	-	Methyl-accepting chemotaxis sensory transducer
CIBE_5656	8up	-1.3	2.5	3.0	2.6	-	Lipopolysaccharide biosynthesis protein
CIBE_5686	8up	-0.6	1.1	3.1	3.2	-	conserved protein of unknown function
CIBE_2493	8up	-0.3	1.8	2.7	2.5	-	Extracellular solute-binding protein, family 3
CIBE_5100	8up	-0.6	1.9	2.6	2.8	ydaM	putative glycosyltransferase associated to biofilm formation
CIBE_4957	8up	0.0	1.9	2.4	2.3	-	conserved protein of unknown function
CIBE_0510	8up	-0.9	1.9	3.0	2.7	-	Endothelin-converting enzyme 1
CIBE_3002	8up	-1.1	1.5	3.5	2.8	-	conserved protein of unknown function
CIBE_3935	8up	-0.3	1.3	2.9	2.7	-	Small, acid-soluble spore protein beta
CIBE_1670	8up	-0.2	1.6	2.8	2.4	-	Alpha-glucosidase 2
CIBE_3149	8up	-0.1	1.4	2.8	2.4	yodR	putative acyloate-acetoacetate CoA-transferase
CIBE_1312	8up	-0.4	0.8	2.8	3.2	-	Autolysin (fragment)
CIBE_4642	8up	0.0	3.3	1.4	1.7	-	conserved membrane protein of unknown function
CIBE_5644	8up	0.0	2.1	2.3	1.9	-	Glycosyl transferase, family 2
CIBE_3150	8up	-0.2	1.4	2.8	2.4	atoA	acetoacetyl CoA-transferase (subunit A)
CIBE_4958	8up	-0.2	1.7	2.7	2.2	-	conserved protein of unknown function
CIBE_0741	8up	0.2	1.8	2.3	2.1	-	conserved protein of unknown function
CIBE_2744	8up	-1.0	2.2	2.9	2.2	-	protein of unknown function
CIBE_1671	8up	0.1	1.6	2.7	2.0	malL	Oligo-1,6-glucosidase
CIBE_2745	8up	-0.7	2.2	3.1	1.7	rbr3A	Reverse rubrerythrin-1
CIBE_0064	8up	0.1	1.8	2.6	1.8	-	conserved protein of unknown function
CIBE_6033	8up	-1.1	1.6	3.1	2.5	-	Phosphohydrolase
CIBE_2316	8up	0.0	2.0	2.2	1.8	-	CarD family transcriptional regulator
CIBE_4956	8up	0.1	1.7	2.3	2.0	-	Amine oxidase
CIBE_1748	8up	-0.8	1.7	3.4	1.7	-	conserved exported protein of unknown function
CIBE_4402	8up	-0.8	1.7	2.6	2.5	-	Dual specificity protein phosphatase
CIBE_5066	8up	0.2	2.2	2.4	1.3	-	protein of unknown function
CIBE_3516	8up	0.1	1.6	2.4	1.9	-	conserved protein of unknown function
CIBE_5436	8up	-0.8	1.6	2.7	2.3	-	Amino acid permease-associated region
CIBE_3517	8up	-0.1	1.6	2.6	1.8	-	Rubrerythrin
CIBE_2560	8up	-0.5	1.1	2.9	2.4	-	conserved exported protein of unknown function
CIBE_2662	8up	-0.1	2.7	2.1	1.0	-	protein of unknown function
CIBE_1668	8up	-0.6	1.6	2.7	2.0	amyD	putative starch degradation products transport system permease protein AmyD
CIBE_5692	8up	-0.7	1.7	2.5	2.3	-	Glycosyl transferase
CIBE_2939	8up	-0.1	1.6	2.3	2.0	-	D-galactose-binding protein
CIBE_3567	8up	0.3	1.8	2.1	1.4	-	Ni/Fe hydrogenase
CIBE_2039	8up	-1.1	1.7	2.6	2.5	spoIVB	regulatory membrane-associated serine protease
CIBE_3924	8up	-0.2	1.5	2.4	2.0	-	Methyltransferase type 11
CIBE_0742	8up	0.1	1.8	2.2	1.6	-	conserved protein of unknown function
CIBE_3256	8up	0.1	1.8	2.1	1.7	-	Sugar transporter
CIBE_1500	8up	-0.5	1.2	3.1	2.0	-	Ferredoxin
CIBE_2196	8up	-0.9	1.1	2.8	2.7	nifJ	Pyruvate-flavodoxin oxidoreductase
CIBE_5643	8up	0.0	1.8	2.3	1.6	-	Glycosyl transferase, group 1
CIBE_4055	8up	-1.1	1.5	2.6	2.7	-	putative galactoside ABC transporter
CIBE_2661	8up	-0.1	2.6	2.0	1.1	-	protein of unknown function
CIBE_1667	8up	-0.9	1.4	2.9	2.2	-	ABC transporter substrate-binding protein
CIBE_4880	8up	-0.6	1.4	2.7	2.2	-	Pyruvate flavodoxin/ferredoxin oxidoreductase domain protein
CIBE_0641	8up	-0.6	0.7	3.0	2.4	ssp	Small, acid-soluble spore protein 1
CIBE_0047	8up	0.0	1.9	2.0	1.4	-	conserved protein of unknown function
CIBE_0738	8up	-0.1	1.3	2.3	1.8	-	conserved protein of unknown function
CIBE_1081	8up	-1.4	1.5	2.7	2.4	-	conserved exported protein of unknown function
CIBE_4034	8up	-0.4	2.1	2.3	1.3	-	conserved membrane protein of unknown function
CIBE_0740	8up	-0.2	1.5	2.2	1.8	rbr	Rubrerythrin
CIBE_5000	8up	0.2	1.7	2.2	1.1	-	conserved protein of unknown function
CIBE_0773	8up	-0.1	1.6	2.4	1.4	-	conserved protein of unknown function
CIBE_4879	8up	-0.5	1.4	2.4	1.9	-	Pyruvate ferredoxin/flavodoxin oxidoreductase, beta subunit
CIBE_1807	8up	-0.2	1.2	2.2	1.9	-	Sporulation protein
CIBE_0231	8up	-0.6	1.3	2.5	1.9	-	conserved protein of unknown function
CIBE_2101	8up	0.1	1.6	2.0	1.3	-	Lactate utilization protein B/C
CIBE_3953	8up	-0.1	1.9	1.6	1.6	-	conserved exported protein of unknown function
CIBE_0739	8up	-0.2	1.3	2.2	1.6	-	Stage V sporulation protein R
CIBE_5688	8up	-0.9	0.4	2.4	3.0	-	conserved protein of unknown function
CIBE_5774	8up	-0.7	1.1	3.0	1.5	-	putative manganese catalase
CIBE_3314	8up	0.0	1.5	2.0	1.4	-	conserved protein of unknown function
CIBE_0737	8up	-0.4	1.2	2.3	1.7	prkA	serine protein kinase
CIBE_2348	8up	-1.7	1.6	3.1	1.8	-	conserved protein of unknown function
CIBE_2879	8up	-0.5	1.0	2.2	2.0	-	Cytochrome b5
CIBE_5642	8up	-0.7	1.7	1.7	1.8	-	Polysaccharide biosynthesis protein
CIBE_2769	8up	-0.3	1.6	2.0	1.3	-	conserved protein of unknown function
CIBE_4807	8up	-0.3	1.6	1.7	1.6	-	exported protein of unknown function
CIBE_0538	8up	-0.3	1.3	1.7	1.8	yabG	sporulation-specific protease
CIBE_2224	8up	-0.1	0.3	2.1	2.2	-	conserved protein of unknown function
CIBE_1771	8up	0.0	1.9	1.6	0.8	-	Membrane protein
CIBE_4865	8up	-1.9	1.8	2.3	2.2	-	Glycosyl transferase
CIBE_5903	8up	-0.9	1.5	2.4	1.4	-	conserved protein of unknown function
CIBE_1043	8up	-0.2	1.4	1.8	1.3	-	Peptidase S8 and S53, subtilisin, kexin, sedolisin
CIBE_3965	8up	-0.6	0.7	2.4	1.7	-	Spore protein
CIBE_4722	8up	-0.5	0.6	2.6	1.6	-	putative galactoside ABC transporter
CIBE_5648	8up	-0.5	1.3	1.7	1.8	-	Glycosyl transferase family 1
CIBE_1232	8up	-0.8	1.0	2.2	1.9	-	Calcium-transporting ATPase 1
CIBE_2198	8up	-0.3	2.4	1.7	0.3	-	protein of unknown function

CIBE_3011	8up	-0.6	1.0	2.4	1.3	-	conserved exported protein of unknown function
CIBE_3600	8up	-0.3	1.4	1.8	1.3	fda	Fructose-bisphosphate aldolase class 1
CIBE_0206	8up	-0.8	0.8	2.0	1.9	-	N-acetylmuramoyl-L-alanine amidase CwlD
CIBE_3070	8up	-0.5	0.5	2.2	1.7	-	Serine acetyltransferase-related protein
CIBE_3462	8up	-0.1	1.5	1.3	1.1	-	Sodium:proton antiporter
CIBE_2293	8up	0.0	0.1	1.7	1.9	-	FAD-dependent oxidoreductase
CIBE_0419	8up	-0.3	1.4	1.4	1.1	nfsB	NAD(P)H-flavin oxidoreductase (nitroreductase)
CIBE_3541	8up	0.1	0.1	1.6	1.8	yrkC	putative dioxygenase; cupin family
CIBE_2065	8up	-0.7	1.3	2.0	0.9	azoR	FMN-dependent NADH-azoreductase
CIBE_4640	8up	-0.1	1.8	1.1	0.7	-	conserved protein of unknown function
CIBE_0807	8up	-1.1	1.1	2.6	1.0	-	conserved protein of unknown function
CIBE_1557	8up	-0.4	1.7	1.5	0.5	-	conserved protein of unknown function
CIBE_4641	8up	-0.1	2.2	0.7	0.6	yvrH	two-component response regulator YvrH involved in cell wall processes [YvrG]
CIBE_5966	8up	0.0	1.6	1.3	0.5	-	conserved protein of unknown function
CIBE_0428	8up	-0.6	1.0	1.9	1.0	-	conserved protein of unknown function
CIBE_5684	8up	-0.7	0.2	2.3	1.5	-	conserved protein of unknown function
CIBE_3428	8up	-0.2	1.5	1.5	0.5	-	conserved protein of unknown function
CIBE_3620	8up	-0.7	1.2	1.6	0.9	-	Uncharacterized NAD(P)H oxidoreductase HI_1544
CIBE_0581	8up	-0.8	1.2	1.8	0.8	-	conserved protein of unknown function
CIBE_2121	8up	-1.0	0.7	2.1	1.1	-	conserved membrane protein of unknown function
CIBE_3649	8up	-1.2	0.5	1.8	1.8	-	Small acid-soluble spore protein, alpha/beta type
CIBE_0042	8up	0.0	1.9	0.8	0.3	-	conserved membrane protein of unknown function
CIBE_1741	8up	-0.5	0.1	1.6	1.8	-	conserved protein of unknown function
CIBE_0040	8up	-0.5	0.0	1.7	1.8	-	Peptidoglycan-binding domain 1 protein
CIBE_1558	8up	-0.3	1.6	1.3	0.4	-	Lysophospholipase
CIBE_3057	8up	-0.8	-0.1	1.6	2.0	-	Glycosyl transferase, family 2
CIBE_0672	8up	-0.2	1.5	0.9	0.5	-	Peptidase M50
CIBE_1544	8up	-0.9	0.9	1.4	1.1	-	Lipase
CIBE_5092	8up	-0.5	1.1	1.2	0.8	-	Sporulation protein YunB
CIBE_0547	8up	-0.8	0.8	1.3	1.2	ispE	4-(cytidine 5'-diphospho)-2-C-methyl-D-erythritol kinase
CIBE_0754	8up	-0.2	1.4	1.0	0.2	mntP	putative manganese efflux pump MntP
CIBE_1646	8up	-0.4	1.2	1.1	0.5	-	Permease
CIBE_1682	8up	-0.7	0.9	1.2	0.7	-	Peptidase S8 and S53, subtilisin, kexin, sedolisin
CIBE_5685	8up	-1.4	0.0	2.2	1.1	-	conserved protein of unknown function
CIBE_2775	8up	-1.3	0.3	1.7	1.3	-	Small, acid-soluble spore protein beta
CIBE_4959	8up	-0.6	1.2	1.2	0.2	mntR	Transcriptional regulator MntR
CIBE_3698	8up	-0.8	-0.1	1.7	0.8	-	Small acid-soluble spore protein, alpha/beta type
CIBE_0673	8up	-0.4	1.3	0.5	-0.1	-	conserved protein of unknown function
CIBE_2216	8up	-0.4	1.2	1.3	-0.9	-	conserved protein of unknown function
CIBE_5060	8up	-0.9	1.1	0.8	0.2	-	Abortive infection protein
CIBE_2258	8down	-0.7	-0.5	-2.3	-2.6	-	conserved protein of unknown function
CIBE_4352	8down	0.1	-2.0	-2.1	-2.8	-	Pyruvate flavodoxin/ferredoxin oxidoreductase domain protein
CIBE_5053	8down	0.6	-1.3	-1.9	-4.3	-	Ferrous iron transport protein B
CIBE_5055	8down	0.4	-1.6	-1.5	-4.9	-	FeoA family protein
CIBE_5054	8down	0.4	-2.1	-1.8	-4.7	-	FeoA family protein
CIBE_1056	11up	-0.9	-0.3	1.8	2.3	yhxC	putative oxidoreductase
CIBE_2329	11up	-0.8	-0.2	1.7	2.1	-	conserved protein of unknown function
CIBE_4882	11up	-0.5	-0.2	1.6	1.7	-	Metallophosphoesterase
CIBE_1066	11up	-0.5	-0.5	1.4	1.9	-	putative ABC transporter, periplasmic-binding
CIBE_5950	11up	-1.0	-0.2	1.7	1.6	-	conserved protein of unknown function
CIBE_0536	11up	-0.9	-0.3	1.3	1.4	-	Glycosyl transferase, group 1
CIBE_0532	11up	-1.4	-0.5	1.5	1.9	-	Glycosyl transferase, group 1
CIBE_5516	11up	-1.0	-0.3	2.1	0.6	tlp	Protein Tlp homolog
CIBE_0533	11up	-1.0	-0.5	1.5	1.4	-	Spore coat protein
CIBE_4006	11up	-1.1	-0.6	1.5	1.6	-	conserved protein of unknown function
CIBE_4742	11up	-1.1	-0.8	1.4	1.5	yisY	AB hydrolase superfamily protein YisY
CIBE_3050	11up	-1.5	-0.6	1.0	1.9	fdtB	dTDP-3-amino-3,6-dideoxy-alpha-D-galactopyranose transaminase
CIBE_0736	11up	-1.5	-0.4	1.3	0.9	-	conserved protein of unknown function
CIBE_3951	11up	-1.2	-0.8	1.4	0.7	-	Spore protein
CIBE_2913	11up	-1.7	-0.2	1.2	0.7	-	Small, acid-soluble spore protein beta
CIBE_1674	11up	-1.3	-0.9	1.2	0.3	-	Small, acid-soluble spore protein beta
CIBE_3604	11up	-2.6	-1.5	1.3	1.5	-	Ferrous iron transport protein B homolog (fragment)
CIBE_tRNA28	11up	-1.7	-0.9	0.1	0.6	-	Gly tRNA
CIBE_4841	11up	-1.6	-0.8	0.1	0.0	buk	branched-chain fatty-acid kinase
CIBE_3605	11up	-3.0	-1.7	0.6	1.0	-	Ferrous iron transport protein B homolog (fragment)
CIBE_3603	11up	-2.9	-1.7	0.2	1.0	-	Iron transporter FeoA
CIBE_4869	11up	-1.5	-2.5	0.2	-0.4	-	Beta-ketoacyl-acyl-carrier-protein synthase I
CIBE_4341	Unclassified	4.7	3.0	1.9	2.1	-	Sugar-binding protein
CIBE_3250	Unclassified	4.2	2.5	1.5	2.3	-	conserved protein of unknown function
CIBE_3258	Unclassified	-1.6	2.4	4.7	3.9	-	PTS maltose transporter subunit IIBC
CIBE_3259	Unclassified	-2.1	1.6	4.4	3.8	-	putative PTS IIA-like nitrogen-regulatory protein PtsN
CIBE_0988	Unclassified	0.1	1.7	0.5	-0.8	-	TspO
CIBE_0920	Unclassified	-0.5	1.3	0.3	-0.2	-	conserved protein of unknown function
CIBE_0080	Unclassified	-0.8	1.0	0.3	0.3	ykoT	putative glycosyltransferase
CIBE_5443	Unclassified	-1.0	0.9	0.3	-0.3	-	Transcription antiterminator BglG
CIBE_0107	Unclassified	1.1	0.8	-1.0	-1.3	-	Sporulation protein
CIBE_0308	Unclassified	1.5	-0.3	-0.7	-0.8	-	Signal transduction histidine kinase regulating citrate/malate metabolism
CIBE_5197	Unclassified	0.7	0.9	-1.1	-1.6	-	conserved exported protein of unknown function
CIBE_0919	Unclassified	-0.8	1.4	-0.6	-1.3	-	protein of unknown function
CIBE_2260	Unclassified	0.0	0.2	-1.5	-1.4	-	conserved protein of unknown function
CIBE_2839	Unclassified	0.2	0.6	-2.1	-1.7	-	protein of unknown function
CIBE_2277	Unclassified	-0.3	0.1	-1.6	-1.2	xkdU	putative phage protein XkdU
CIBE_0350	Unclassified	-4.1	-4.7	-1.5	-1.4	guaB	inosine-monophosphate dehydrogenase
CIBE_2740	Unclassified	-1.2	-3.2	-4.6	-5.0	treA	trehalose-6-phosphate hydrolase
CIBE_2739	Unclassified	-1.0	-3.4	-5.3	-5.1	treP	phosphotransferase system (PTS) trehalose-specific enzyme IIBC component
CIBE_0034	Unclassified	-3.2	-4.6	-6.5	-4.5	-	conserved protein of unknown function
